# Rad51–Rad52 Mediated Maintenance of Centromeric Chromatin in *Candida albicans*

**DOI:** 10.1371/journal.pgen.1004344

**Published:** 2014-04-24

**Authors:** Sreyoshi Mitra, Jonathan Gómez-Raja, Germán Larriba, Dharani Dhar Dubey, Kaustuv Sanyal

**Affiliations:** 1 Molecular Mycology Laboratory, Molecular Biology and Genetics Unit, Jawaharlal Nehru Centre for Advanced Scientific Research, Jakkur, Bangalore, India; 2 Departamento Ciencias Biomédicas Área de Microbiología, Universidad de Extremadura, Badajoz, Spain; 3 Department of Biotechnology, VBS Purvanchal University, Jaunpur, India; Duke University, United States of America

## Abstract

Specification of the centromere location in most eukaryotes is not solely dependent on the DNA sequence. However, the non-genetic determinants of centromere identity are not clearly defined. While multiple mechanisms, individually or in concert, may specify centromeres epigenetically, most studies in this area are focused on a universal factor, a centromere-specific histone H3 variant CENP-A, often considered as the epigenetic determinant of centromere identity. In spite of variable timing of its loading at centromeres across species, a replication coupled early S phase deposition of CENP-A is found in most yeast centromeres. Centromeres are the earliest replicating chromosomal regions in a pathogenic budding yeast *Candida albicans*. Using a 2-dimensional agarose gel electrophoresis assay, we identify replication origins (*ORI7-LI* and *ORI7-RI*) proximal to an early replicating centromere (*CEN7*) in *C. albicans*. We show that the replication forks stall at *CEN7* in a kinetochore dependent manner and fork stalling is reduced in the absence of the homologous recombination (HR) proteins Rad51 and Rad52. Deletion of *ORI7-RI* causes a significant reduction in the stalled fork signal and an increased loss rate of the altered chromosome 7. The HR proteins, Rad51 and Rad52, have been shown to play a role in fork restart. Confocal microscopy shows declustered kinetochores in *rad51* and *rad52* mutants, which are evidence of kinetochore disintegrity. CENP-A^CaCse4^ levels at centromeres, as determined by chromatin immunoprecipitation (ChIP) experiments, are reduced in absence of Rad51/Rad52 resulting in disruption of the kinetochore structure. Moreover, western blot analysis reveals that delocalized CENP-A molecules in HR mutants degrade in a similar fashion as in other kinetochore mutants described before. Finally, co-immunoprecipitation assays indicate that Rad51 and Rad52 physically interact with CENP-A^CaCse4^ *in vivo*. Thus, the HR proteins Rad51 and Rad52 epigenetically maintain centromere functioning by regulating CENP-A^CaCse4^ levels at the programmed stall sites of early replicating centromeres.

## Author Summary

The epigenetic mark of centromeres, CENP-A, is deposited in S phase in most yeasts by a mechanism that is not completely understood. Here, we identify two *CEN7* flanking replication origins, *ORI7-L1* and *ORI7-RI*, proximal to an early replicating centromere (*CEN7*) in a budding yeast *Candida albicans*. Replication forks starting from these origins stall randomly at *CEN7* by the kinetochore that serves as a barrier to fork progression. We observe that centromeric fork stalling is reduced in absence of the HR proteins, Rad51 and Rad52, known to play a role in restarting stalled forks. Further, we demonstrate that Rad51 and Rad52 physically interact with CENP-A^CaCse4^ *in vivo*. CENP-A^CaCse4^ levels are reduced in absence of Rad51 or Rad52, which results in disruption of the kinetochore structure. Here we propose a novel DNA replication-coupled mechanism mediated by HR proteins which epigenetically maintains centromere identity by regulating CENP-A deposition. A direct role of DNA repair proteins in centromere function offers insights into the mechanisms of centromere mis-regulation that leads to widespread aneuploidy in cancer cells.

## Introduction

The centromere (*CEN*) is a specialized chromosomal locus that recruits a macromolecular multi-protein complex, called the kinetochore that binds to spindle microtubules and helps in equal separation of chromosomes during the anaphase stage of mitosis. Despite performing a conserved function, *CEN* DNA sequences are highly variable. In most eukaryotes inheritance of centromeric chromatin is regulated epigenetically by an atypical chromatin structure marked by a centromere specific variant of histone H3, called as CENP-A [1], [2]. Centromeres constitute a distinct replication timing domain during S phase [3]. Centromeric chromatin has been observed to replicate during early S phase in diverse unicellular organisms such as budding yeasts *Saccharomyces cerevisiae* [4] and *Candida albicans* [5], fission yeast *Schizosaccharomyces pombe* [6] and protozoan *Trypanosoma brucei* [7]. Interestingly, the loading of CENP-A is replication coupled in *S. cerevisiae* [8], [9] whereas the loading is biphasic (S and G2) in *S. pombe* [10]–[12].

The physical proximity of centromeres and replication origins appears to be evolutionarily conserved in single celled organisms - prokaryotes and unicellular yeasts. In bacteria such as *Bacillus subtilis* and *Vibrio cholerae*, the centromere like *parS* loci are always proximal to the unique chromosomal origin *ori*C [13]. *CEN*s are associated with one or more early firing origins in many yeast species. In an industrial yeast *Yarrowia lipolytica*, each centromere is associated with a proximal origin mapping within 1 kb of the centromere [14]. Even in fission yeast *S. pombe*, replication origins are clustered in the dg and dh repeats surrounding the central core [15], [16]. Proximity of *CEN*s to early replicating origins (*ORI*s) raises the question whether replication plays a direct role in regulating centromere location and function and/or *vice versa*. In bacteria, the *parS* binding protein ParB has been observed to regulate replication initiation from *oriC* as well as to recruit structural maintenance of chromosome (SMC) proteins at *parS* to promote efficient segregation of the bacterial genome [17]. Early replication timing of centromeres appears to be important for proper kinetochore assembly in *S. cerevisiae* [18]. On the other hand, active mechanisms have been observed in both *S. cerevisiae* and *S. pombe* by which centromeres control early replication of pericentric origins [5], [16], [19], [20]. The heterochromatic protein Swi6, a homolog of mammalian HP1 in fission yeast, has been found to regulate the early initiation of pericentric origins [16]. However, in absence of a conserved HP1 ortholog in *S. cerevisiae*, the Ctf19 kinetochore complex performs a similar function [20].

It has been reported that replication forks stall at yeast centromeres [15], [21]. The constitutive presence of a kinetochore at *CEN*s in *S. cerevisiae* forms a bi-directional protein-DNA barrier that stalls replication forks approaching from either direction [21], [22]. Fork stalling signals have also been identified at *CEN*s in another budding yeast *Y. lipolytica* [14] and fission yeast *S. pombe* [15]. A variety of cellular mechanisms are known to stabilize the replication machinery at the stalled sites or restart the collapsed replication forks from these stalls. Specialized helicases have been known to relieve fork stalling in *S. cerevisiae* and *S. pombe* protein-DNA barriers, in a non-recombinogenic manner [23], [24]. However, in certain natural fork stalling sites homologous recombination (HR) has been involved in restarting fork movement [25]–[27]. Two HR proteins Rad51 and Rad52, which are traditionally involved in the repair of double strand breaks (DSBs), have been shown to bind transiently during S phase to unperturbed replication forks [28] and also at site-specific protein-DNA barriers [26]. Further Rad52 has also been shown to bind at stabilized stalled forks in a Smc5/6 dependent manner where it catalyzes nascent strand exchange required for fork restart by both Rad51-dependent and -independent mechanisms [29]. These results suggest a possible involvement of HR proteins Rad51 and Rad52 at protein-DNA barriers during normal S phase. Recently an interesting link between replication fork stalling and centromere functioning was identified in the members of the constitutive centromere associated network (CCAN), CENP-S and CENP-X, which are conserved between yeast and humans [30]. Apart from their essential roles in kinetochore assembly, these proteins also aid in the processing of stalled or blocked replication forks in a recombination dependent manner [31]. Finally, Rad51 was observed to bind to *CEN*s during S phase in *S. pombe*, where it prevented isochromosome formation [32].

The short regional *CEN*s of the pathogenic diploid budding yeast *Candida albicans* have 3–5 kb CENP-A binding region comprised of unique sequences [33], [34] without characteristic centromere-specific sequence motifs or pericentric repeats. The boundaries of these *CEN*s are ill-defined since functional stable centromeric plasmids could not be constructed and centromere formation was found to be epigenetically regulated [35]. In the absence of any sequence requirement, the *CEN* DNA has diverged rapidly not only between two closely related *Candida* species [36], but also among evolutionarily distant clinical strains of *C. albicans* [37]. However, the location of the centromere is found to be conserved for several million years [36], [38]. Further, on deletion of the native centromere, *C. albicans* can efficiently form neocentromeres proximal to the native centromere [38], [39]. Thus chromosomal location, rather than the DNA sequence *per se*, appears to be an important determinant for centromere formation. The maintenance of centromere location, in absence of any obvious pericentric boundary, through each cell cycle remains a puzzle. However, replication timing microarray studies demonstrated that both centromeres and neocentromeres in *C. albicans* replicate earliest in S phase and each *CEN* is flanked by the earliest firing origin in the genome [5]. Although it was proposed that distinct replication timing could be a regulator for epigenetic maintenance of *C. albicans CEN*s, no active mechanism has been suggested.

We recently demonstrated that gene conversion can be an active mechanism to restore centromere location in *C. albicans* [38]. One possibility is that naturally occurring gene conversion-associated mutagenesis [40] drives accelerated change in *CEN* DNA sequences.

Considering these lines of circumstantial evidence as described above, we first sought to determine how centromere associated origins may regulate centromere functioning. We demonstrate that replication forks pause at *C. albicans* centromeres by the functional kinetochore. Deletion of a centromere proximal origin impacts the fidelity of chromosome segregation. We also show that centromeric fork stalling is reduced in the absence of HR proteins Rad51 and Rad52. Finally, we provide evidence revealing roles for Rad51 and Rad52 in recruiting the centromeric histone CENP-A to the centromeres which can epigenetically maintain centromeric chromatin in *C. albicans*. Overall our study describes a novel circuitry between three major biological processes – DNA replication, DNA recombination-repair and chromosome segregation - that orchestrates faithful propagation of genetic material to the next generation.

## Results

### Replication forks stall at *C. albicans* centromeres

**Figure 1:**
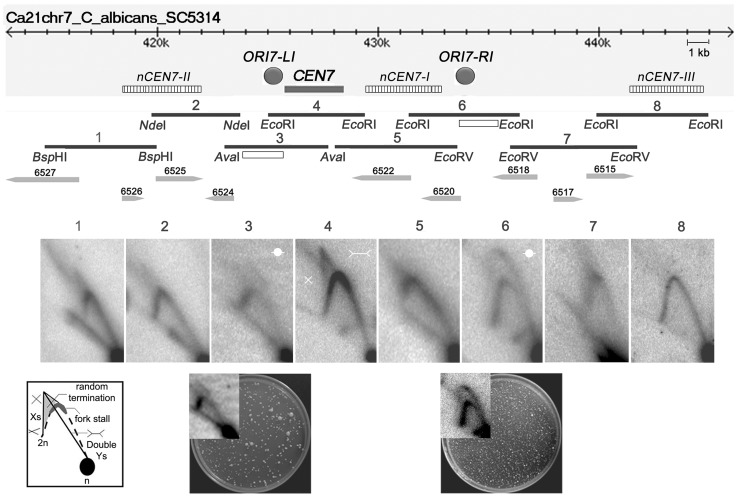
Replication forks stall/terminate randomly during centromere replication. Schematic of a ∼30 kb region of chromosome 7 centered on the centromere (*CEN7*) is shown. The hatched rectangles denote the positions of the nearest neocentromere (*nCEN7*) hotspots as described earlier [38]. The filled grey circles indicate the positions of the chromosomal origins identified during 2D analysis (*ORI7-LI* and *ORI7-RI*). Replication intermediates from this 30 kb region in asynchronously grown *C. albicans* cells were analyzed by 2D gel electrophoresis assays using overlapping restriction fragments (1–8). Arrowheads and numbers indicate the positions and the identities of the ORFs. Open rectangles indicate the fragments used for the *ARS* function assay. Schematic of replication intermediates indicates simple ‘Y’ arcs (broken line), specific termination (Double-Ys), joint molecules (Xs) and random termination signals (triangular smear). The dark grey zone at the inflection point of the ‘Y’ arc indicates replication fork stalling. The presence of Xs and triangular smear in fragments 1, 2, 4, 5 and 7 indicates replication fork stalling/termination. Bubble arcs are observed in fragments 3 and 6 signaling chromosomal origins of replication (*ORI7-LI* and *ORI7-RI*). The plate pictures in the lower panel show the results of an *ARS* function assay using the fragments (open rectangles) located within *ORI7-LI* and *ORI7-RI* in the wild-type. The corresponding 2-D signals in high contrast are shown in the inset. Both fragments show *ARS* activity.

To determine the progress of replication through *C. albicans CEN*s, neutral-neutral 2 dimensional agarose gel electrophoresis assays were performed and the replication intermediates in and around the centromere of chromosome 7 (*CEN7*) were characterized. For that purpose, we analyzed overlapping restriction fragments (3–5 kb length) covering the ∼3 kb *CEN7*, ∼12 kb upstream and ∼17 kb downstream regions of *CEN7* (Figure 1). The core CENP-A^CaCse4^ binding region of *CEN7* (fragment 4) showed the presence of ‘simple Y’ arcs but no ‘bubble’ arc, an indication that the centromere was passively replicated by origins lying outside the region. A ‘cone-shaped’ signal was observed above the Y arc. Previous studies [41] have shown that such cone signals are composed of ‘double-Y’ specific termination intermediates, a smeary triangular zone of ‘random termination’ and ‘X’ spikes or joint molecules (schematic in Figure 1). Fork stalling at *S. cerevisiae* ‘point’ *CEN*s were observed as distinct spots on the Y arc [21]. However, random fork pausing over a broad region of highly transcribed genes (such as the RNA polymerase II genes) was observed as the complex cone-signal instead of discrete Y arc spots [42]. Therefore, occurrence of the cone-signal indicated that replication forks stalled/terminated randomly over the ∼3 kb CENP-A^CaCse4^ binding region of *CEN7*. Apart from the random termination signal, an accumulation of signals was observed at the apex of the Y arc (fragment 4 and fragment 5 in Figure 1, Figure S1A and S1B), which indicated fork stalling in this region. Fork stalling at a specific site within the *CEN*s allows time for replication forks to enter from the other end of the fragment and converge at the stall sites, resulting in termination. Immediately upstream of *CEN7* a faint bubble arc signal was observed in an overlapping restriction fragment (fragment 3 in Figure 1), indicating the presence of an active replication origin. This origin is termed as *ORI7-LI*. A zone of random termination was observed further upstream in the fragments 1 and 2. On analyzing the *CEN7* downstream, approximately 3 kb away from the core *CEN7* (fragment 4 in Figure 1) an *Eco*RI fragment (fragment 6 in Figure 1) was found to contain a bubble arc indicating the presence of a second chromosomal origin of replication, which was named as *ORI7-RI*. Origin-less plasmids carrying fragments spanning these origin containing regions (open rectangles in Figure 1 line diagram) showed high frequency transformation of *C. albicans* indicating that these origin regions possessed autonomously replicating sequence (*ARS*) activity (plate photographs in Figure 1).

Upon observing fork stalling at *CEN7* we wanted to test whether this is a generalized event across *C. albicans CEN*s. The centromere of chromosome 5 (*CEN5*) is unique among *C. albicans CEN*s in having the longest inverted repeats in the pericentric region (∼2.2 kb) [37]. The 2-D analysis revealed fork termination and stalling at the core *CEN5* region whereas characteristic origin-specific bubble signals were observed in the immediate upstream and downstream regions (*ORI5-LI* and *ORI5-RI*) (Figure S1C). The positions of *ORI5-LI* and *ORI5-RI* are proximal to neocentromere hotspots (*nCEN5-I* and *nCEN5-II*) [38] on chromosome 5 (Figure S1D). A *CEN5* proximal origin has been described previously in the *CEN5* upstream region [5]. Thus general features of *C. albicans* centromeres include random fork termination at the core CENP-A^CaCse4^ binding region and presence of flanking active origins.

### A centromere-proximal origin maintains centromere functioning

**Figure 2:**
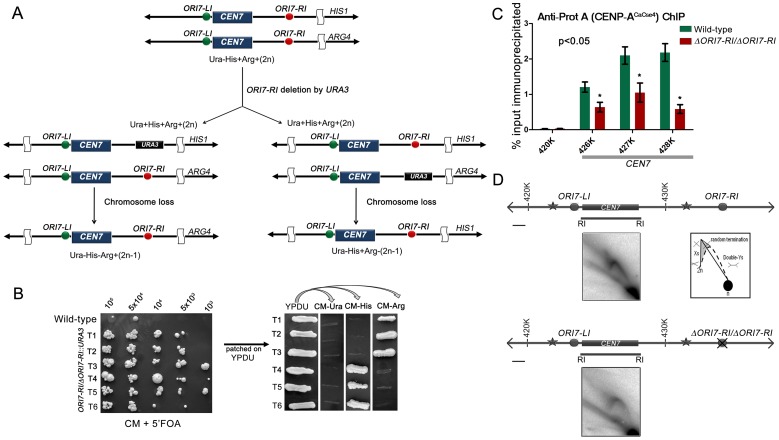
Centromere proximal origins maintain centromere functioning. (A) Schematic showing the strategy of deletion of *ORI7-RI* (shown as red circles) and the chromosome loss assay. Since the strain RM100AH [33] is heterozygous for both *HIS1* and *ARG4* that mark the two chromosome 7 homologs, replacement of *ORI7-RI* by *URA3* will create a strain that can be used to assay the loss of the altered chromosome (by scoring simultaneous loss of Ura and Arg or Ura and His markers). (B) Dilutions of *ORI7-RI* deleted transformants (*ORI7-RI/*Δ*ORI7-RI*) were spotted on CM+5′-FOA plates to estimate the chromosome loss frequency. USN148 (Δ*ura3::imm434/*Δ*ura3::imm434*/CIp10) strain was used as the wild-type control to estimate the spontaneous loss rate of a chromosome. Subsequently, the 5′-FOA positive colonies were patched on YPDU. From YPDU these colonies were re-patched onto CM- Ura, CM-His and CM-Arg plates to assay for the loss of the altered chromosome 7 homolog. (C) Standard ChIP assays followed by quantitative real time PCR (qPCR) were performed in wild-type and CAKS105 (Δ*ORI7-RI/*Δ*ORI7-RI*) strain for enrichment of CENP-A^CaCse4^-Prot A at the core *CEN7*. Enrichment of CENP-A^CaCse4^ at the centromere was calculated as a percentage of the total chromatin input and values were plotted as mean of two independent experiments (three technical replicates for each experiment) ± SD. (D) Line diagrams depicting ∼15 kb region surrounding *CEN7* are shown (symbols as in Figure 1). Schematics depict the replication intermediates as described in Figure 1. Bar (black line), 1 kb. The upper panel shows the 2-D image from the core *CEN7* region (fragment 4 in Figure 1) in the wild-type. The lower panel shows the 2-D image from the same fragment when *ORI7-RI* is deleted in CAKS105 (Δ*ORI7-RI/*Δ*ORI7-RI*).

A previous study demonstrated that *C. albicans* centromeres are the earliest replicating regions in the genome and formation of neocentromeres led to the activation of an origin in an adjacent locus [5]. The study concluded that early replication could be an epigenetic mechanism for maintaining centromere position. In this study, we sought to test whether replication properties of pericentric regions can regulate centromere function by deleting one of the two *CEN7*-proximal origins. Deletion of a single copy of *ORI7*-*RI* formed the strain CAKS104 (*ΔORI7-RI::URA3/ORI7-RI*) which showed a moderate loss of the altered chromosome 7 (∼5×10^−3^ to 1×10^−3^) (Figure 2A and 2B). This loss rate was comparable to a *CEN* deleted chromosome stabilized by the formation of a neocentromere [38]. Further, both copies of *OR17-RI* were deleted to generate a homozygous *ORI7*-*RI* deletion strain CAKS105 (*ΔORI7*-*RI::URA3/ΔORI7*-*RI::NAT*). CENP-A^CaCse4^-Prot A ChIP followed by qPCR in CAKS105 revealed that the levels of CENP-A^CaCse4^ enrichment over the ∼3 kb *CEN7* are significantly reduced as compared to the wild-type (Figure 2C). Thus, both a higher rate of chromosome loss and a reduced enrichment of CENP-A^CaCse4^ at *CEN7* of the altered chromosome indicate that centromere fitness is reduced upon deletion of a *CEN* proximal origin.

Deletion of an active origin of replication often changes the replication dynamics of a broad region [43]. We were curious to know how deletion of *ORI7*-*RI* affected replication fork movement in or around *CEN7*. The 2D gel analysis of the core *CEN7* region (fragment 4 in Figure 1) in CAKS105 (*ΔORI7*-*RI/ΔORI7*-*RI*) strain revealed the similar pattern of Y arc indicating passive replication of the centromere. However, there was a clear absence of the random termination signal which was observed in the wild-type. In addition, the stall signal at the apex of the Y arc appeared to be reduced in CAKS105 (Figure 2D). Thus deletion of a *CEN* proximal origin resulted in the loss of the random termination signal at the centromere which exhibited compromised function. These findings propelled us to investigate whether there was an active mechanism by which fork stalling/termination helps in centromere functioning in *C. albicans*.

### Fork stalling at the centromere is kinetochore mediated and involves the HR proteins Rad51 and Rad52

**Figure 3:**
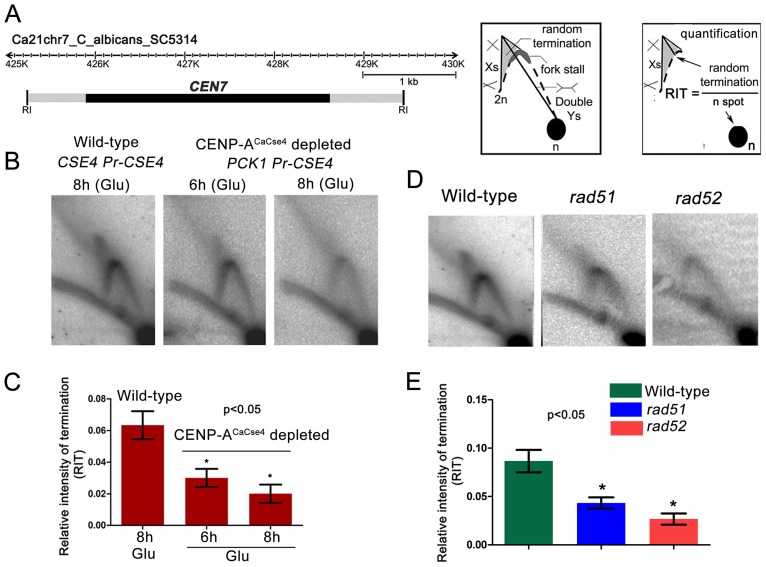
Centromeric fork stalling/termination is CENP-A mediated and involves Rad51 and Rad52. (A) A line diagram of a 6 kb region of chromosome 7 centered on *CEN7* is shown. *CEN7* (black rectangle) and flanking regions (grey rectangles) that include the 5 kb *Eco*RI fragment (fragment 4 in Figure 1) used for 2D gel analysis are shown. Schematics of replication intermediates as described in Figure 1 are also shown. Quantification of the termination signals was performed as following: Relative intensity of termination (RIT) = random termination signal/1n spot. (B) Replication intermediates from the core *CEN7* region were determined by 2-D gel analysis at wild-type and depleted levels of CENP-A^CaCse4^. (C) The 1n spot (schematic) and the termination signals (triangular smear) were quantified by Image Gauge software (Fujifilm) and RIT values were calculated as described above for wild-type and CENP-A^CaCse4^ depleted condition. The RIT values, plotted on a bar graph, indicate a gradual decrease in the termination signal in CENP-A^CaCse4^ repressed conditions as compared to wild-type. The values represent the mean of three independent 2D experiments ± SD. (D) Replication intermediates from the core *CEN7* region (black rectangle) were determined by 2D gel analysis for the wild-type, *rad51*, and *rad52* mutants. (E) RIT values were calculated for wild-type and *rad51* and *rad52* mutants. The RIT values, plotted on a bar graph, indicate a decrease in the termination signal in *rad51* and *rad52* mutants as compared to wild-type. The values represent the mean of three independent 2D experiments ± SD.

Previous studies from *S. cerevisiae* indicated kinetochore-mediated fork stalling at *CEN*s [21]. Based on this information, we decided to test whether an active kinetochore acts as a barrier to replication fork movement by depleting CENP-A^CaCse4^. Depletion of CENP-A^CaCse4^ leads to a loss of kinetochore integrity, affecting the localization of many other key kinetochore proteins [44]. The strain CAKS3b (*cse4*/*PCK1*pr-*CSE4*) [36] was grown overnight in succinate (overexpression), transferred to the repressive YPDU media and cells were harvested after 6 h or 8 h of growth. Genomic DNA was isolated from these cells and replication intermediates of the 3 kb core *CEN7* region (Figure 3A) was analysed by 2-D gel assays. Corresponding 2-D DNA blot from the wild-type (*CSE4/CSE4*) cells grown for 8 h in YPDU served as the control (Figure 3B). The intensities of the cone-shaped signal and the 1n spot were measured in each blot and the relative intensity of termination (RIT) was calculated after background correction (Figure 3C). Similarly, the intensities of the stall signal and the 1n spot were measured in each blot and the relative intensity of stall (RIS) was calculated (Figure S2). The 2D experiments were repeated thrice and mean RIT and RIS values were computed along with standard deviation. RIT values indicated a 3-fold reduction in termination (Figure 3C) whereas RIS values showed a reduction of ∼6-fold after 8 h of CENP-A^CaCse4^ depletion as compared to the wild-type (Figure S2). Overall, this reduction in intensity indicates a kinetochore mediated fork stalling mechanism at the centromere. On observing a CENP-A^CaCse4^ mediated fork stalling at the centromere, we speculated that if the HR proteins, Rad51 and Rad52, are involved at these sites of fork-stalling, we would observe a reduction in the termination/stall signal in absence of these proteins as well. We tested this hypothesis by probing the core *CEN7* region using 2D gel analysis in wild-type, *rad51* and *rad52* mutants (Figure 3D). A ∼3 fold reduction of the termination signal (RIT) was observed in the *rad52* mutant, whereas the reduction was ∼2 fold in absence of Rad51 (Figure 3E). The fork stalling signal (RIS) was reduced by ∼4 times in *rad52*, whereas it was reduced by about ∼3 times in *rad51* (Figure S2). These results indicated involvement of Rad51/Rad52 in fork stalling at *C. albicans* centromeres.

### Rad51 or Rad52 depletion results in a failure of kinetochore assembly

In *C. albicans*, the effects of Rad51 and Rad52 have been studied mainly through their deletion mutants [45], [46]. Rad52 depletion leads to large bud arrest (80%) and a 100-fold higher rate of loss of heterozygosity (LOH) than the wild-type [47]. Most of LOH arises due to chromosome loss or truncation. Similar effects have been observed under Rad51 depletion (Larriba G. unpublished). A plausible explanation for this high chromosome loss rate may be a dysfunctional centromere in absence of Rad52, leading to increased chromosome non-disjunction. Based on these reported facts, we were curious to know whether the recombination proteins had a role to play in centromere function, apart from or associated with their role in fork restart. To test this hypothesis, we examined whether *rad51* or *rad52* mutant exhibits hallmarks of centromere dysfunction including loss of kinetochore integrity and/or reduced or mis-localization of essential kinetochore proteins [44].

**Figure 4:**
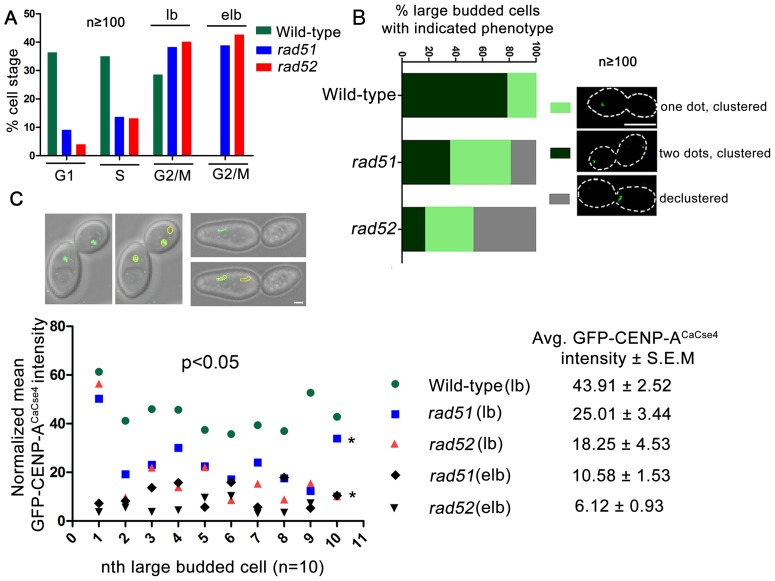
Rad51 or Rad52 depletion affects kinetochore assembly. (A) Percentage of cells at each cell cycle stage was determined for wild-type, *rad51* and *rad52* mutants. At least 100 cells were counted at each stage. lb, large bud; elb, extended large bud. The extended large bud (elb) is an aberrant G2/M phenotype observed in *rad51* and *rad52* mutants only. (B) Using confocal microscopy, GFP-CENP-A^CaCse4^ foci were scored in large budded cells of wild-type, *rad51* or *rad52* mutant strains. They were classified into three categories as shown in figure. n≥100. The percentage of large budded cells under each category was calculated for wild-type and mutant strains, and plotted. An increase in percentage of large budded cells with declustered GFP-CENP-A^CaCse4^signals is observed in *rad51* or *rad52* mutant, which is an indicator of improper kinetochore assembly [44]. Bar (white line), 5 µm. (C) Intensity of the GFP-CENP-A^CaCse4^ spots was measured by the Image J software for wild-type, *rad51* or *rad52* mutant cells for the G2/M stage, n = 10 in each case. The normalized mean GFP intensity (with respect to background) was calculated for each cell and plotted. Legend shows the different categories of strains and stages and the average GFP-CENP-A^CaCse4^ intensity ± S.E.M. Associated DIC images show the measurement technique for calculating GFP intensity. Bar (white line), 1 µm.

In order to visualize the kinetochore organization under Rad51 or Rad52 depletion conditions, both copies of *RAD51* or *RAD52* were deleted in a strain where one copy of CENP-A^CaCse4^ was GFP tagged [48]. The kinetochore integrity was monitored through studying GFP-CENP-A^CaCse4^ localization by confocal microscopy. Kinetochores are tightly clustered in *C. albicans*, showing a single CENP-A^CaCse4^ dot-like signal per bud throughout the cell cycle [49]. In the event of a kinetochore dysfunction, such as reduced protein levels and/or delocalization of essential kinetochore proteins, the kinetochores become declustered, showing multiple (2 or more) dots of CENP-A^CaCse4^ or a ‘stretch’ of several weak CENP-A^CaCse4^foci [44]. Initially, the percentage of cells in different cell cycle stages (G1, S and G2/M) was determined in wild-type, *rad51*, and *rad52* mutants. Cell counting showed that a significant percentage of cells in *rad51* and *rad52* strains were arrested at the G2/M stage (large bud and extended large bud) (Figure 4A). Large budded (G2/M) cells were scored for the GFP- CENP-A^CaCse4^ foci in wild-type, *rad51*, and *rad52* mutant strains. There was a significant decrease in the percentage of normal large budded cells in *rad51* (36.4%) and *rad52* (17.3%) as compared to wild-type (78.6%) (Figure 4B). Further, there was an increase in the percentage of large budded cells with single GFP-CENP-A^CaCse4^ dot in *rad51* and *rad52* mutants that indicates a checkpoint arrest or delay at the S or G2/M stage of the cell cycle. Finally, a significant fraction of large budded cells had declustered GFP-CENP-A^CaCse4^ foci in *rad51* (18.7%) and *rad52* (47.1%) mutants. The declustered GFP-CENP-A^CaCse4^ foci indicate aberrant localization of the protein, resulting in defective kinetochore architecture [44].

Declustering was also confirmed by indirect immunolocalization of CENP-A^CaCse4^-Prot A foci using anti-Prot A antibodies (Figure S3). While studying the CENP-A^CaCse4^ localization we observed, both by GFP fluorescence and antibody localization, a reduction in the signal intensity of CENP-A^CaCse4^, especially at the G2/M stage. In order to quantify the loss, GFP- CENP-A^CaCse4^ intensity was measured at different stages of the cell cycle. A significant loss of intensity was observed at the G2/M stages in *rad51* (reduced by ∼43%) and *rad52* (reduced by ∼58%) as compared to the wild-type (Figure 4C and Figure S4A and D), whereas the levels are similar in unbudded and small budded cells (Figure S4A–D). Prolonged depletion of essential kinetochore proteins like CENP-A^CaCse4^ and Mis12^CaMtw1^ results in an extended large bud phenotype [44], [50]. Deletion of *RAD51* or *RAD52* also showed similar cells where the intensity of GFP-CENP-A^CaCse4^ was severely diminished (Figure 4C and S4A). Thus, both in terms of the kinetochore integrity and CENP-A^CaCse4^ localization, *rad51* and *rad52* mutants depicted significant defects, as compared to wild-type. However, the percentage of declustering and the decrease in GFP-CENP-A^CaCse4^ intensity was enhanced in *rad52* as compared to *rad51*.

To further confirm the kinetochore dysfunction observed in *rad51* and *rad52* mutants, localization pattern of the middle kinetochore protein Mis12^CaMtw1^ [50] was studied by indirect immunofluorescence using anti-Prot A antibodies in a Prot A-tagged Mis12^CaMtw1^ strain in the wild-type and *rad51* or *rad52* mutants. Mis12^CaMtw1^ localization also showed a similar pattern, with the G2/M cells of *rad51* and *rad52* showing declustering of the Mis12^CaMtw1^-Prot A foci as opposed to the clustered localization observed in the wild-type cells (Figure S5).

### Rad51–Rad52 complex aids in CENP-A recruitment

**Figure 5:**
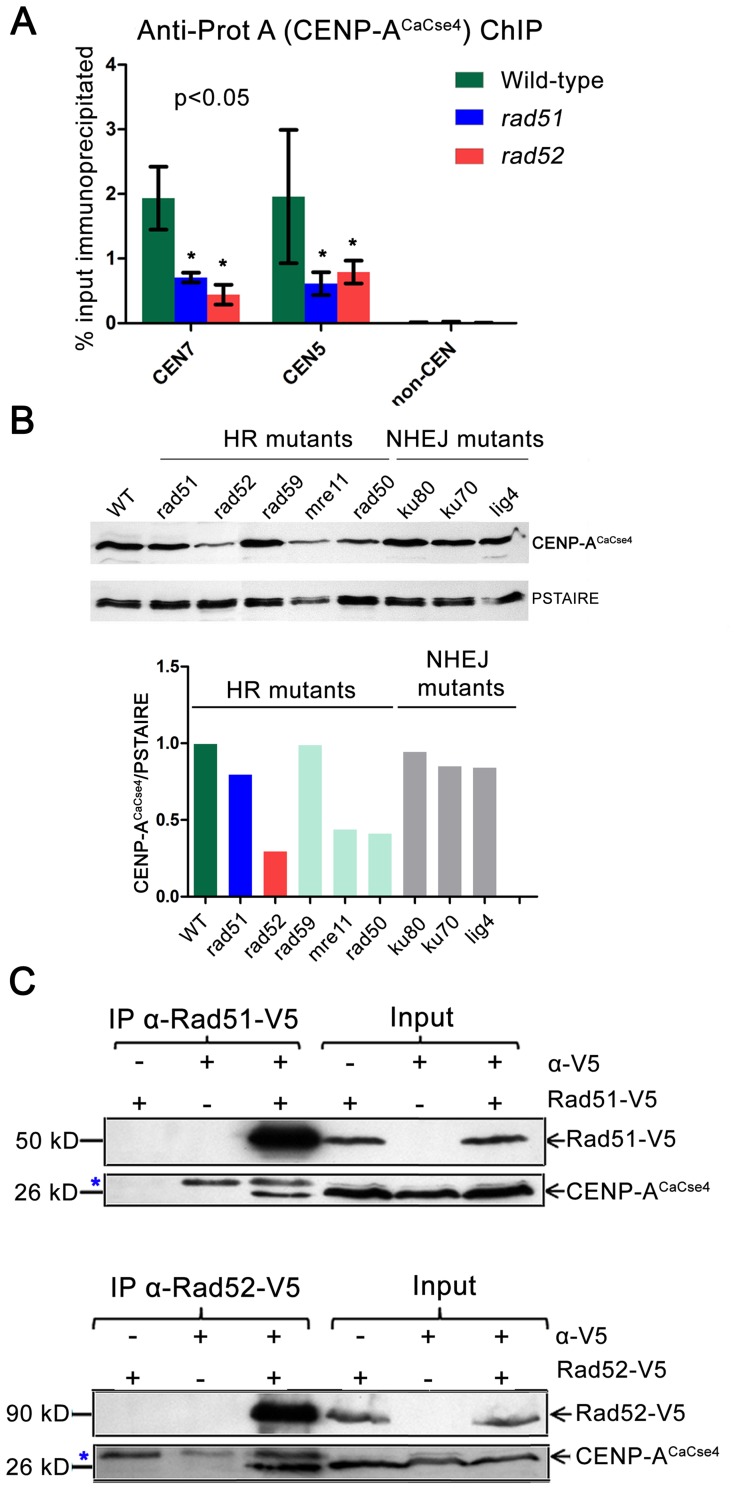
Rad51 and Rad52 aid in CENP-A^CaCse4^ recruitment. (A) Standard ChIP assays followed by quantitative real time PCR (qPCR) were performed in wild-type, *rad51* or *rad52* for CENP-A^CaCse4^-Prot A for *CEN5* and *CEN7*. qPCR amplification from a non-centromeric (*non-CEN*) control was also performed to detect the background DNA elution in the ChIP assays. Enrichment of CENP-A^CaCse4^ at the centromeres was calculated as a percentage of the total chromatin input and values were plotted as mean of three independent ChIP experiments ± SD. (B) Western blot analysis performed with the whole cell lysates from wild-type and homologous recombination (HR) and non-homologous end joining (NHEJ) mutants using anti-CENP-A^CaCse4^ antibodies. PSTAIRE was used as a loading control. The relative levels of CENP-A^CaCse4^ (CENP-A^CaCse4^/PSTAIRE) was computed for each mutant and plotted in a bar graph. (C) Co-immunoprecipitation assays for the two sets of strains carrying Rad51-V5 and Rad52-V5 were performed using anti-V5 antibodies. Precipitates were analyzed by western blotting with anti- CENP-A^CaCse4^ antibodies. In each case, untagged strains and no (-) antibody fractions were used as controls. Blue asterisk indicates a non-specific band.

One of the mechanisms through which the Rad51–Rad52 complex can regulate kinetochore assembly is through participation in the CENP-A^CaCse4^ recruitment at the centromere. A series of circumstantial evidence indicate that repair and CENP-A deposition may be intricately connected. Induction of specific double strand breaks was shown to recruit CENP-A to break sites in mammalian cell lines [51]. Besides, in *Xenopus* eggs, base excision repair (BER) proteins were required for establishment of CENP-A chromatin [52]. To investigate a possible link between the Rad51–Rad52 complex and CENP-A deposition, the enrichment levels of CENP-A^CaCse4^ at *C. albicans CEN*s were studied by ChIP assays using anti-Prot A antibodies against CENP-A^CaCse4^-Prot A in the wild-type, *rad51* and *rad52* mutants. Quantitative real time PCRs (qPCR) were performed in triplicate with *CEN5* and *CEN7* specific primers using the immunoprecipitated DNA from three independent ChIP assays. Relative enrichment was computed as percentage of the total chromatin input. Approximately 2 and 3 fold reductions in CENP-A^CaCse4^ enrichment were observed across the *CEN*s in *rad51* and *rad52* strains, respectively (Figure 5A). Similarly, ChIP followed by qPCR was performed to calculate the enrichment of Mis12^CaMtw1^-Prot A at *CEN5* and *CEN7* under similar conditions. Mis12^CaMtw1^ binding was also found to be reduced in *rad51* or *rad52* mutants as compared to the wild-type (Figure S6A).

On finding reduced binding of kinetochore proteins to the *CEN*s in absence of Rad51 or Rad52, we next addressed the fate of delocalized CENP-A^CaCse4^ or Mis12^CaMtw1^ proteins in the cell. Western blots were performed with whole cell lysates using anti-CENP-A^CaCse4^ antibodies to detect the levels of CENP-A^CaCse4^ in wild-type, *rad51* and *rad52* strains. CENP-A^CaCse4^ levels were reduced in *rad51* as compared to the wild-type whereas the levels were further diminished in *rad52* strain (Figure 5B). However, levels of Mis12^CaMtw1^-Prot A remained unaltered across wild-type and mutant strains (Figure S6B). Further, we also studied the levels of CENP-A^CaCse4^ in the absence of other repair proteins. The CENP-A^CaCse4^ levels remained unchanged in the absence of the Rad52 paralog Rad59 as well as members of the non-homologous end joining (NHEJ) pathway Ku70, Ku80 and Lig4 (Figure 5B). However, CENP-A^CaCse4^ levels were depleted in absence of Mre11 and Rad50, proteins that are involved in the initial processing of DSBs (Figure 5B). In order to determine the mode of CENP-A^CaCse4^ regulation by Rad51 and Rad52, *CSE4* RNA levels were estimated by quantitative reverse transcription PCR (qRT-PCR) from wild-type, *rad51* and *rad52* cells. The qRT-PCR results indicated a marginal reduction in *CSE4* RNA levels in *rad52* as compared to the wild-type whereas the RNA levels remained unchanged in *rad51* (Figure S6C). Previously, we had shown that expression of a non-degradable CENP-A^CaCse4-7R^ restores the CENP-A levels in kinetochore mutants. To observe whether a similar rescue is a possibility in HR mutants, *RAD51* and *RAD52* were deleted in the non-degradable CENP-A^CaCse4-7R^ strain (*cse4/CSE47R-TAP*) [44] to get GRC409 (*cse4/CSE47R-TAP;rad51/rad51*) and GRC425 (*cse4/CSE47R-TAP; rad52/rad52*). Western blot with anti- Prot A antibodies showed an increased level of CENP-A^CaCse4-7R^-Prot A in *rad51* as compared to the level of CENP-A^CaCse4^-Prot A in the same mutant (Figure S6D). Thus, expression of a non-degradable CENP-A rescues CENP-A protein levels in the *rad51* mutant, suggesting that unincorporated CENP-A^CaCse4^ is destroyed by the proteasome mediated degradation in this strain. However, similar replacement of CENP-A^CaCse4^-Prot A by CENP-A^CaCse4-7R^ failed to completely rescue the CENP-A^CaCse4^ levels in *rad52* mutant (Figure S6D) probably due to a reduced transcription of the *CSE4* gene (Figure S6C).

Recruitment of CENP-A^CaCse4^ by Rad51 and Rad52 would implicate that they would be part of a supramolecular complex. In order to study a possible *in vivo* interaction between these proteins, coimmunoprecipitation experiments were performed separately with Rad51-V5 and Rad52-V5 strains. Immunoprecipitates obtained by anti-V5 antibodies were analyzed by western blotting with anti-CENP-A^CaCse4^ antibodies [49]. CENP-A^CaCse4^ was pulled down by both Rad51-V5 and Rad52-V5, indicating an interaction between CENP-A^CaCse4^ and Rad51, and CENP-A^CaCse4^ and Rad52 (Figure 5C). Finally, ChIP was performed with anti-V5 antibody in unsynchronized cells of Rad51-V5 and Rad52-V5 followed by qPCR with *CEN7* specific primers in order to find whether Rad51 and Rad52 were enriched at *C. albicans* centromeres. ChIP-qPCR results from three independent experiments indicated a significant enrichment of Rad51 and Rad52 at *CEN7* as compared to a non-*CEN* region present ∼100 kb upstream of *CEN7* (Figure S6E).

## Discussion

The mechanism of CENP-A chromatin establishment remains elusive in the epigenetically regulated centromeres of *C. albicans*. In this study we investigated the role of DNA replication and repair in this process. We show that replication forks stall at *C. albicans* centromeres in a kinetochore dependent manner. Deletion of a *CEN*-proximal active origin results in reduced fork stalling as well as compromised centromere function of the altered chromosome. Incidentally, fork stalling/termination at the centromere are also reduced in absence of Rad51 or Rad52. These two proteins are shown to be involved in kinetochore assembly.

Conservation of the centromere location is a common feature in closely related *Candida* species [36], [38]. Neocentromere ‘hotspots’ have been shown to be localized within 10–15 kb of the native centromere [38], [39]. Hence chromosomal location has been implicated as a determinant for centromere/neocentromere identity. Although a genome-wide study had shown that each *C. albicans* centromere is associated with an early firing origin [5], the precise origin locus had not been mapped. Using 2-D gel analysis we have mapped the nearest origins to *CEN5* and *CEN7* within ∼5 kb *CEN* flanking regions. Further, it was shown that neocentromere formation led to activation of new origins in its vicinity, in *C. albicans* [5]. In this study, we show that deletion of a *CEN* proximal origin results in reduced centromere activity. Interestingly, both *ORI7-LI* and *ORI7-RI* are proximal to established neocentromere hotspots *nCEN7-I* and *nCEN7-II* respectively ([38] and Figure 1 line diagram). Deletion of a 4.5 kb region including *CEN7* leads to the formation of *nCEN7-I* and *nCEN7-II*. The 4.5 kb deletion leaves ∼1.2 kb of the upstream intergene which is part of the *ORI7-LI*. However, on deletion of the entire 6.5 kb ORF-free region surrounding *CEN7* (which deletes entire *ORI7-LI*) only the *nCEN7-I* hotspot remains and a new hotspot *nCEN7-III* is observed downstream of *ORI7-RI* (Figure 1 line diagram and [38]). Thus the formation of *nCEN7-II* appears to be correlated with the presence of the proximal origin *ORI7-L1*. This raises an attractive possibility that the position of centromere/neocentromere formation in *C. albicans* is governed by the proximity of sequences having potential or active origin properties. Analysis of replication intermediates in and around all reported centromere/neocentromere locations by 2-D gel assays can further confirm this hypothesis.

**Figure 6:**
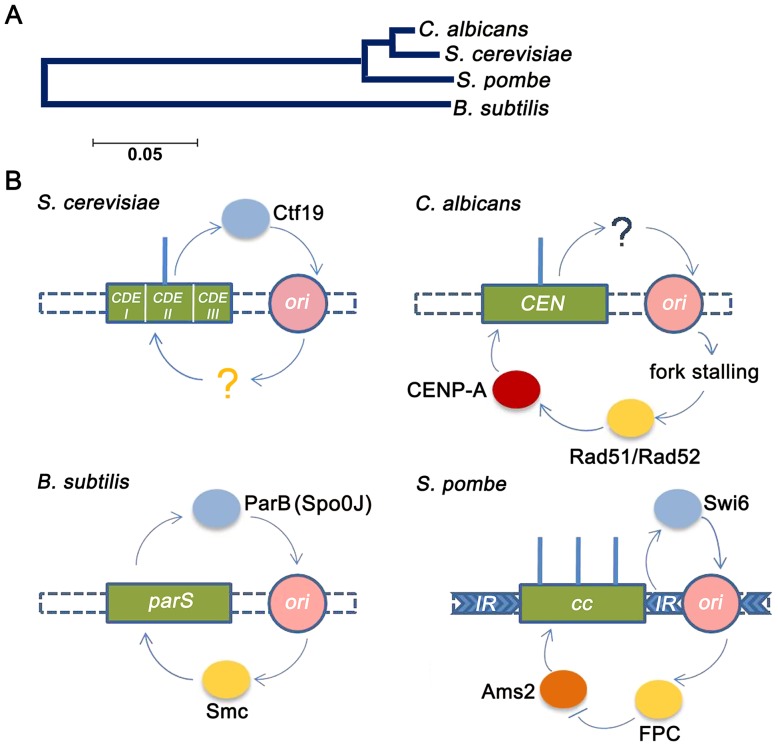
Replication-segregation interaction is evolutionarily conserved in unicellular organisms. (A) The phylogenetic tree reflects the evolutionary relationships of the corresponding taxa. The tree is drawn to scale with branch lengths in the units of the number of base substitutions per site in the 23S or 25S rRNA nucleotide sequences of the four species. (B) *CEN*-like loci or *CEN*s (green boxes) in prokaryotes and unicellular eukaryotes respectively are flanked by early replication origins (pink circles). The blue circles indicate the centromere factors influencing origin activity. The yellow circles indicate the origin/replication associated factors influencing *CEN* function. In the genome of the bacteria *B. subtilis*, the single replication origin is flanked by *CEN*-like *parS* sequence. The Spo0J (ParB) protein, binding to *parS*, organizes *ori* activity as well as recruits Smc proteins for proper segregation [17]. In *S. cerevisiae*, which has short ‘point’ centromeres, the Ctf19 complex directly recruits initiation factors for early firing of proximal origins [20]. Although early firing has been suggested for playing a role in *CEN* function, no *cis* factors has been identified. In *C. albicans*, which has ‘short regional’ *CEN*s, *CEN*s have been shown to govern early replication of proximal origins, although no *cis* factors were identified [5]. In this study we show that fork stalling at *CENs* from proximal origins recruit Rad51/Rad52 that, in turn, regulates CENP-A deposition. Finally in the ‘large regional’ centromeres of *S. pombe*, the centromeric heterochromatic protein Swi6 activates pericentric replication origins [16]. The fork protection complex (FPC) that travels with the replisome negatively regulates Ams2 [73] that, in turn, regulates CENP-A deposition [10].

The functional significance of these interactions, however, may not be limited to the linear chromosomal level [3]. For example, bacterial origins of replication show asymmetric association with old and new poles, and mid region of the cell during replication [53]. Similarly, centromeric clustering has been predicted to facilitate kinetochore formation by increasing the local availability of kinetochore proteins [38], [44]. In chicken DT40 cells it has been shown that on centromere deletion, residual CENP-A in the pericentric region favours the formation of neocentromeres in close proximity to the native locus [54]–[56]. However, our studies in *C. albicans* have shown that even on deletion of a ∼30 kb flanking region of the *CEN7*, neocentromere formation was found to be proximal to the deleted region [38]. This result posits a paradigm that factors apart from the local concentration of CENP-A molecules such as chromatin conformation, CENP-A loading and replication timing, and/or proximal origins may govern centromere/neocentromere establishment. Thus, this study in conjunction with previous reports suggest an evolutionarily conserved relationship between *CEN* DNA replication timing and origin-centromere proximity at least in unicellular organisms - both in bacteria and yeasts (Figure 6A and 6B).

As observed previously in kinetochore protein mutants [44], [50], [57], the *rad51* and *rad52* mutants showed characteristic features of an improper kinetochore structure including increased kinetochore declustering, loss of essential kinetochore proteins from the centromere and CENP-A degradation via a proteasome mediated pathway. Specifically, the effect of Rad52 has been found to be more pronounced than Rad51 at stalled forks [26], [28], indicating that Rad52 binding at stalled forks may have additional repair independent roles that are nevertheless important for maintaining the stability and integrity of these stall sites. Emerging views on the role of repair proteins at the centromeres suggest that they are primarily involved in stabilization/protection of stalled replication forks while deleterious end results of HR such as cross-over recombination or gross chromosomal rearrangements are carefully prevented [58]. Further, studies have shown that stabilization of stalled replication forks contribute to the stability of pericentromeric heterochromatin [59]. Therefore, a repair independent role of Rad52 at the inner centromere may be envisioned in preserving kinetochore integrity by regulating pericentromeric heterochromatin maintenance. However, the reduction in CENP-A levels in *mre11* and *rad50* strains, in addition to *rad52*, suggests that a repair-dependent role of Rad52 may also be a possibility at the centromere. Based on earlier observations [44], the observed reduction in the level of CENP-A in *rad51* or *rad52* mutant is probably due to the degradation of CENP-A that is not properly recruited to the centromeres in absence of these proteins. Although this seems to be true for the *rad51* mutant, we find that *CSE4* transcription is also reduced in the *rad52* mutant. Since a global change in the transcription has been observed in the *rad52* mutant (G. Larriba, unpublished), it is possible that reduction in *CSE4* transcript may be a secondary effect which is adding to the overall decrease in the CENP-A protein level observed in our experiment.

The physical localization of CENP-A and Rad51/Rad52 in a complex is a strong indicator of a HR protein mediated CENP-A recruitment. An indirect evidence from the mammalian system shows that the HR machinery can be directly involved in CENP-A loading at the centromeres. HJURP, the cell cycle specific CENP-A chaperone, has been observed to work downstream of the ATM pathway that recognises and mediates repair of DSBs by HR in cancerous tissues [60]. The homolog of HJURP has been identified in *S. cerevisiae* and *S. pombe*, where it is known as Scm3 [61].

**Figure 7:**
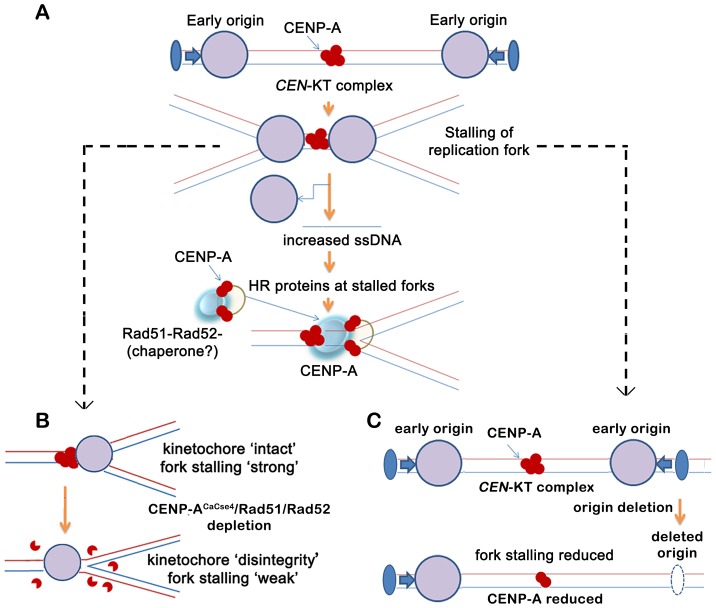
A replication-coupled repair based model of centromere inheritance. (A) A replication-coupled repair based model for propagation of CENP-A^CaCse4^ chromatin at an early replicating centromere. During S phase, replication forks, originating from proximal conserved early origins, stall at the kinetochore. The stalling of replication forks at the centromere leads to accumulation of single stranded (ss) DNA. The homologous recombination proteins Rad51 and Rad52 are possibly recruited via ssDNA to the stalled replication forks at the centromere. A transient Rad51/Rad52-CENP-A^CaCse4^ complex is stabilized by one or more cell-cycle regulated proteins (chaperone?) at the centromere, thereby regulating the replication coupled deposition of CENP-A at the centromeres. The CENP-A^CaCse4^ bound kinetochore is indicated by red circles whereas replisome is depicted by large purple circle. Functional origin locations are shown by blue filled ovals. *CEN*- centromere, KT- kinetochore. (B) Schematic depicts the effect of CENP-A^CaCse4^/Rad51/Rad52 depletion on replication fork passage through the centromere. Depletion of CENP-A^CaCse4^ causes kinetochore disintegrity. As a result forks are no longer stalled at the *CEN*-kinetochore barrier and fork stalling is weakened. Depletion of Rad51/Rad52 also causes improper kinetochore assembly. As a result fork stalling is weakened. (C) Schematic depicts the effect of deletion of a proximal origin on replication fork passage through the centromere. On deletion of a proximal origin, fork stalling at the centromere is reduced and concomitantly CENP-A^CaCse4^ binding is reduced, leading to a weaker centromere.

Combining the observations from our experiments and previous studies, we propose a hypothesis for a replication coupled repair mediated loading of CENP-A and maintenance of CENP-A chromatin at *C. albicans CEN*s (Figure 7A–C). The forks from the centromere-proximal early firing origins are initially blocked by the pre-existing CENP-A (the kinetochore complex). The presence of single-stranded, non-linear DNA regions (fork, termination or both) for sufficiently long time (ensured by early fork arrival) triggers a transient recruitment of Rad51/Rad52 proteins. Experiments in mammalian cells have shown that CENP-A has a propensity to bind to DSBs and probably aids in DSB repair [51]. Based on this observation and our co-immunoprecipitation results we propose that CENP-A at least transiently, remains in a complex with Rad51/Rad52. Perhaps presence of a chaperone like HJURP/Scm3, which can also bind to Holliday junctions, stabilizes the CENP-A brought in by Rad51/Rad52, thereby ensuring its *CEN* only deposition. Crucial to this hypothesis is the requirement of a cell-cycle regulated intermediate that can convert a general association between the repair proteins and CENP-A to facilitate a Rad51/Rad52 mediated recruitment of CENP-A to the *CEN*s. While a cell-cycle regulated chaperone is a plausible factor, other possible candidates may include proteins like the transcription factor Ams2 [10] that promote the centromeric localization of CENP-A during S phase in *S. pombe*. Interestingly, the S phase specific expression of Ams2 was found to be regulated by Hsk1 which also interacts with the replication fork protection complex (FPC) [62], [63] (Figure 6B).

However, this Rad51/Rad52 mediated mechanism may not be the only mode of CENP-A deposition at *C. albicans* centromeres. In fact, non-lethality of *rad51* or *rad52* mutants in *C. albicans*, despite their important kinetochore function, indicates that there are overlapping pathways that, independently or in conjunction, may be important for establishment of CENP-A chromatin. Nevertheless, in humans ‘hotspots’ for neocentromere formation has been mapped to regions of inverted duplication that are prone to breakage and repair [64]. Therefore a repair-mediated pathway appears to be conserved in evolution as a mechanism for epigenetic propagation of centromere chromatin.

## Materials and Methods

### Strains and growth conditions

The yeast strains and plasmids used in this study are listed in Table S1. The primers used in this study are listed in Table S2. The *C. albicans* strains were grown in yeast extract/peptone/2% dextrose (YPD) supplemented with uridine (0.1 mg/ml), yeast extract/peptone/2% succinate (YPS) supplemented with uridine or supplemented synthetic/dextrose (SD) minimal media as described previously. *NAT1* containing strains were grown on plates containing nourseuthricin (Nat) at a concentration of 100 µg/ml. The 5-fluoro-oritidic acid (5′-FOA) concentration used in the chromosome loss experiment was 1 µg/µl.

For CENP-A^CaCse4^ depletion assays, wild-type BWP17 (*CSE4* Pr-*CSE4*) cells were grown for 8 h in YPDU. The mutant CAKS3b (*cse4*/*PCK1* Pr-*CSE4*) strain was grown overnight in YPSU (CENP-A^CaCse4^overexpression), transferred to YPDU (CENP-A^CaCse4^ repression) and grown for 6 or 8 h. Genomic DNA was isolated from these cells and replication intermediates from the core *CEN7* region were analyzed by 2-D gel assays.

### Strain construction

*RAD51* and *RAD52* were disrupted as previously described [65] using the *ARG4* and the *HIS1* marker and the primers RAD51D-F and –R and RAD52D-F and –R for amplifying the upstream and downstream regions of *RAD51* and *RAD52* genes, respectively. Correct transformants were identified by PCR using the primers RAD51det-F, -R1, RAD52det-F, -R1, ARG4det-R and HIS1det-R. Rad51 and Rad52 expressing C-terminally tagged V5 epitope (strains GRC68 and GRC83, respectively) were generated by transformation with V5-*URA3* cassettes amplified by PCR with primers RAD51F2/RAD51R1 and RAD52F2/RAD52R1 and pMG2090 as template. Correct integrations were identified by PCR using the primers RAD51det-F1, -R3, RAD52det-F1, -R3, and V5det-R. Sequential disruption of both alleles of *RAD52* in the YJB8675 (CSE4/*CSE4-GFP*) [48] background was performed as described before [45] using the *URA* blaster method. The strain was verified by confirmatory PCR using the primers SM-1 and SM-2. The strain CAKS102 [*CSE4/CSE4-TAP (URA3)*] was constructed by using a C-terminal Prot A tagging cassette using long primers SM-42 and SM-43 in the wild-type SN148 background. CAKS103 [*CSE4/CSE4-TAP (HIS1)*] was constructed by replacing the *URA3* marker in CAKS102 with the *HIS1* replacement cassette using the primers SM36-41. CAKS106 (*MTW1/MTW1-TAP*) was constructed by integrating a C-terminal Prot A tagging cassette pMTU2 [50] in a wild-type SN148 background. Deletion of single copy of *ORI7-RI* was performed with a *URA3* deletion cassette constructed by overlap extension PCR using the primers SM-26-31 in the strain RM1000AH [33]. The resulting strain CAKS104 was confirmed by Southern hybridization with a probe amplified by the primers 2498-15 and 2498-24. Sequential deletion of both copies of *ORI7-RI* was performed in the strain CAKS103 using *NAT* and *URA* deletion cassettes constructed by overlap extension PCR using the primers SM 26-35. The resulting strain CAKS105 was confirmed by Southern hybridization with a probe amplified by primers 2498-15 and 2498-16. USN148 was constructed by integrating the CIp10 plasmid [66] containing Ca*URA3* gene at the RPS10 locus in SN148 background.

### Protein preparation and western blot analyses

Protein extraction was performed as follows: overnight grown cultures were diluted 200-fold into fresh YPDU broth and grown at 30°C for 6 h. Cell pellets were resuspended in ice-cold RIPA buffer (50 mM Tris pH 8, 150 mM NaCl, 1% NP-40, 3 mM EDTA, 0.5% deoxycholate, 0.1% SDS, 10 mM DTT) containing the protease inhibitor cocktail (Sigma) and lysed with acid-washed glass beads (Sigma) in the FastPrep FP120 (Thermo) for 30 s (speed 6.0) twice. Lysates were cleared twice by centrifugations at 4°C to remove cell debris and protein concentration was determined using a spectrophotometer.

For western blot assays, around 4 µg of total protein was diluted in 2X SDS gel loading buffer, boiled at 95°C for 3 min and run in 6–17% SDS polyacrylamide gel electrophoresis (SDS-PAGE). Gels were transferred to a nitrocellulose membrane and blocked in 6% nonfat milk in TBS-T. Membranes were incubated with a 1∶5000 dilution of anti-V5 (Invitrogen Cat. No R960-25), anti-Protein A (Sigma Cat. No P3775) anti-CaCse4 [49] or anti-PSTAIRE (Sigma Cat. No P7962), or 1∶1000 dilution of anti-Act1 (Invitrogen) in 6% non-fat milk TBS-T. Membranes were washed 3 times in TBS-T and then exposed to a 1∶1000 dilution of either anti-mouse- or anti-rabbit -horseradish peroxidase antibody (Pierce) in 6% nonfat milk in TBS-T. Membranes were washed 3 times in TBS-T, incubated with SuperSignal West Dura Extended Duration Substrate (Pierce), and exposed to X-ray films. Band intensities obtained on the autoradiogram were quantified using Adobe Photoshop.

### Co-immunoprecipitation

V5-tagged versions of Rad51 and Rad52 were immunoprecipitated using 10 µl of anti-V5 and 100 µl of Protein A-Sepharose 4B beads (Sigma). Beads were incubated with antibodies for 1 h, added to 0.8 ml of crude extracts (around 3 mg of total protein) and incubated overnight at 4°C. Beads were washed four times with TBS-T. Proteins were eluted by resuspension of beads in 10 µl of 2x SDS gel loading buffer and incubation at 95°C for 5 min for analysis by western blot.

### Indirect immunofluorescence

*C. albicans* cells were grown till OD_600_ of 1 and were fixed by 37% formaldehyde at room temperature. Antibodies were diluted as described: 1∶1000 for rabbit anti-Protein A (Sigma Cat. No P3775), 1∶500 for Alexa Fluor 568 Goat anti-rabbit IgG (Invitrogen). The positions of nuclei were determined by DAPI staining. Cells were examined at 100× magnification on a confocal laser scanning microscope (LSM 510 META, Carl Zeiss). Microscopic images were captured using LSM 510 META software with following lasers for specific flurophores: He/Ne laser (bandpass 565–615 nm) for Alexafluor 568 and a 2-photon laser near IR (bandpass∼780 nm) for DAPI. Palette adjustment was performed to obtain optimal intensity for each image. Z-stacks were collected at 0.4–0.5 µm intervals and stacked projection images were processed in Adobe Photoshop.

### Cytological analysis of GFP-tagged strains

GFP-CENP-A^CaCse4^ strains were grown in YPDU overnight for pre-inoculum, and inoculated in fresh YPDU at an A_600_ of 0.02. The cells were allowed to grow till A_600_ of 1. Then the cells were pelleted down and washed thrice with sterile distilled water. Harvested cells were resuspended in sterile distilled water and representative GFP images were captured with the help of confocal microscope (LSM 510 META, Carl Zeiss). Ar laser (bandpass 500–550 nm) with Z sectioning at 0.4–0.5 µm intervals was applied to scan GFP signals. Images were further processed by Adobe Photoshop software. GFP spots on each nucleus were counted in large budded cells from the images taken.

### Measurement of fluorescence intensities of CENP-A^CaCse4^–GFP signals

ImageJ software (NIH) was used to measure the fluorescent intensities of CENP-A^CaCse4^-GFP signals. Using the appropriate selection tool the area covering the accumulation of the brightest GFP signals (kinetochores) was selected (oval/elliptical selection in case of clustered kinetochores and free-hand selection in case of declustered kinetochores) in each cell. The average pixel intensity in this region was determined and corrected for background by subtracting the lowest pixel intensity value of an area of the same size within the cell. Measurements were taken from 10 cells at each stage under wild-type and mutant conditions. Images for all the cells were collected under identical conditions and contrast adjusted equally. Error values were calculated as standard error of the mean (S.E.M) for the total number of cells. One-way ANOVA and Bonferroni post test analysis was performed to calculate statistical significance.

### Chromatin Immunoprecipitation (ChIP)

Chromatin immunoprecipitation (ChIP) followed by PCR analysis was done as described previously [33]. Rabbit anti-Protein A antibodies (Sigma Cat. No P3775) was used for ChIP at a final concentration of 20 µg ml^−1^ of immunoprecipitate (IP). Asynchronous cultures of *C. albicans* strains were grown in YPDU till A_600_ of 1.000. They were cross-linked with 37% formaldehyde for 15 min (for CENP-A^CaCse4^-Prot A), 30 mins (for Mis12^CaMtw1^-Prot A) and 110 min (for Rad51/Rad52-V5). Subsequently, sonication was performed with Biorupter (Diagenode) to get sheared chromatin fragments of an average size of 300–500 bp. The fragments were immunoprecipitated with anti-Protein A antibodies (Sigma) and anti-V5 antibodies (Invitrogen). ChIP DNA was analyzed by qPCR using primer pairs that amplify central regions of *CEN5* (CACH5F1/CACH5R1) and *CEN7* (nCEN7-3/nCEN7-4). In addition, two other primer pairs (nCEN7-1/nCEN7-2 and nCEN7-5/nCEN7-6) were used for ChIP-qPCR in CAKS105. Amplification from a non-centromeric control region was also performed to detect the background immunoprecipitated DNA. For anti-Protein A ChIP analysis, qPCR was performed on a Rotor Gene 6000 realtime PCR machine with IQ Sybr Green Supermix (Bio-Rad). Cycling parameters were as follows: 94°C/30 s, 55°C/30 s, 72°C/45 s repeated 40×. Melt curve analysis was performed from 55°C to 94°C. Error bars were calculated as standard deviation for three technical replicates of each ChIP sample from at least two independently grown cultures.

### ChIP-qPCR enrichment calculation

The CENP-A^CaCse4^ enrichment was determined by the percent input method. In brief, the Ct values for input were corrected for the dilution factor and then the percent of the input chromatin immunoprecipitated by the antibody was calculated as 100×2^(Adjusted Input Ct- IP Ct)^. One way ANOVA and Bonferroni post tests were performed to determine statistical significance.

### RT-PCR and RT-qPCR

Total RNA was extracted as previously described [67]. Then, RNA was treated with DNase I (Thermo) and the RNA concentration was determined spectrophotometrically. One microgram of total RNA was reverse transcribed to cDNA using Maxima First Strand cDNA Synthesis Kit for RT-qPCR (Thermo). For both RT-PCR and RT-qPCR, one microliter was used as the template with *CSE4*-specific primers (CSE4-F and –R, and qCSE4-F and –R, respectively) and *CDC28*-specific primers (CDC28-F and –R, and qCDC28-F and –R, respectively) as the loading control. RT-PCR parameters were 95°C for 2 min. followed by 30 cycles of 95°C for 30 sec., 53.6°C for 30 sec. and 72°C for 1 min, and a final step of 72°c for 7 min. RT-qPCR parameters were as follows: 95°C for 10 min. and 40 cycles of 95°C for 15 sec. and 59°C for 1 min. Melt curve was performed from 60 to 95°C. 2 Fold increase to *CDC28* was calculated by the Comparative Ct Method. Results are derived from 2 independent experiments with samples by triplicates. Error bars were calculated as standard deviation for the two independent experiments.

### Two dimensional agarose gel electrophoresis of DNA replication intermediates

Two dimensional (2D) agarose gel electrophoresis of DNA replication intermediates was performed as described previously [68]. Briefly, high quality genomic DNA was extracted from log-phase *C. albicans* cells by the CsCl density centrifugation method and digested with suitable restriction enzymes. Benzoylated Napthoylated DEAE (BND) cellulose fractionation was performed on this digested DNA to enrich for single stranded DNA. The enriched DNA was loaded onto 0.4% agarose gel in 1X TBE buffer and run for 15–16 h at 15–20 volts. After run in the first dimension, the desired lanes were cut and run in the second dimension (at 90° to the first) in 1.1% agarose gel in 1X TBE for 2–4 h at 100–125 volts in presence of EtBr (0.3 mg/ml). After that, Southern blotting was done under alkaline transfer conditions. The blots were hybridized with probes from the corresponding restriction fragments. Hybridized membranes were exposed to Phosphorimager films and images captured by Phosphorimager using the Image Reader FLA5000 Ver.2 software.

### Quantification of 2-D image signals

Quantification of the random termination signal (triangular smear) and the pause signal was performed using the Quant option of Image Gauge software version 4.0 (Fujifilm). Quantification in all these cases was done on the raw unprocessed image using the Phosphorimager Image Quant software taking care that the signals are not saturated. For quantitation of RI signals, the signal from 1n spot (unsaturated) is compared with the RI signal of the particular RI type. In each case the area is drawn manually and kept constant for background deduction of all comparisons. For example, the stalled-fork signal area is identical for the wild-type and CENP-A-depleted cells (6 h & 8 h) (Figure 3B). Same is the case with the termination signal. Experiments are repeated and precautions, as much as possible with 2D gels, have been taken to avoid variability. Quantitation is an average of at least two independent DNA preparations. Relative intensity of termination (RIT) was calculated as followed: RIT = normalized random termination/normalized 1N. Similarly, the relative intensity of stall (RIS) was calculated. One way ANOVA and Bonferroni post tests were performed to determine statistical significance.

### ARS plasmid assay

Initially a 1.3 kb Ca*URA3* sequence amplified from the genome was cloned into the *Hind*III site of pUC19 to generate the plasmid pKS101 (Table S1). A 1.4 kb intergenic region from fragment 6 (open rectangle, *ORI7-RI*) and a 2.4 kb intergenic region from fragment 3 (open rectangle, *ORI7-L1*) in Figure 1 were cloned in pKS101 to form the plasmids p*ORI7-RI* and p*ORI7-LI* respectively. Equal amounts of these plasmids (∼1 µg) were transformed into *C. albicans* BWP17 strain using the spheroplast transformation method, as described previously [69] and plated onto supplemented synthetic/dextrose (SD) minimal media without uridine. Transformant plates were incubated at 30°C for 5 days and plate pictures were taken using a digital camera (Nikon COOLPIX 8800VH).

### 5′ FOA-mediated chromosome loss assay

Cells were grown in non-selective liquid medium (YPDU) to an OD_600_ of ∼1, and two and five fold serial dilutions (10^5^, 5×10^4^, 10^4^ etc.) were spotted onto CM+5′- FOA (1 µg/µl) and YPDU plates. The plates were incubated for 4 days at 30°C. FOA resistant colonies were then carefully picked up, streaked on YPDU plates and grown for 1 day. From YPDU these cells were transferred to CM-Ura, CM-Arg and CM- His plates. Plates were incubated for 4 days at 30°C and photographed.

### Phylogenetic tree construction

The evolutionary history was inferred using the Neighbor-Joining method [70]. The tree is drawn to scale, with branch lengths in the same units as those of the evolutionary distances used to infer the phylogenetic tree. The evolutionary distances were computed using the Maximum Composite Likelihood method [71] and are in the units of the number of base substitutions per site. The analysis involved 4 nucleotide sequences of the 23S (*B. subtilis*) and 25S (*S. cerevisiae*, *C. albicans* and *S. pombe*) rRNA gene sequences. Evolutionary analyses were conducted in MEGA5 [72].

## Supporting Information

Figure S1 Analysis of fork stalling at *C. albicans* centromeres. (A) Schematics of replication intermediates as described in Figure 1. High contrast images of fragments 4, 5 and 8 of Figure 1 are reproduced in order to visualize the enhanced *CEN7* stall signals. The stall signal is marginally expanded towards the ascending portion of the Y arc in fragment 5 as compared to fragment 4 (area bordered by white dotted lines). The blot containing fragment 4 when reprobed for fragment 8 does not show the intense signal at the inflection of the Y arc. (B) Three independent 2-D blots of the fragment 5 are shown in order to demonstrate the shift in the stall signal. (C) A line diagram of ∼10 kb region of chromosome 5 centered on the centromere (*CEN5*) is shown. The grey rectangle indicates *CEN5*. Restriction fragments (black lines 1–3) covering the upstream, core *CEN5* and downstream regions are shown. On probing wild-type *C. albicans* genomic DNA (digested with the specified enzyme and separated by 2-D gel electrophoresis) with a unique region within *CEN5*, the 2-D blot shows the presence of both Y arcs as well as termination signals (white arrow in 2). Upstream and downstream fragments 1 and 3 show the presence of bubble arcs (white arrow in 1 and 3), indicating origins. (D) A line diagram of a ∼28 kb region around *CEN5* along with the position of the ORFs (grey arrowhead). The corresponding positions of *CEN5* and the nearest neocentromeres, *nCEN5-I* and *nCEN5-II*, are shown. Similarly, the positions of the origins *ORI5-LI* and *ORI5-RI* are indicated. The arrowheads (dark brown) represent the inverted repeats surrounding *CEN5*.
(TIF)

Figure S2 Fork stalling at *C. albicans* centromeres involves CENP-A^CaCse4^ and Rad51/Rad52. Quantification of the stall signal was performed as following: Relative intensity of stall (RIS) = fork stalling signal/1n spot. The 1n spot (schematic) and stall signals (intensity at the inflection of Y arc) were quantified by Image Gauge software (Fujifilm) and RIS values were calculated as described previously for wild-type vs CENP-A^CaCse4^ and wild-type vs Rad51/Rad52 depleted condition. The RIS values, plotted on a bar graph, indicate a gradual decrease in the stall signal from wild-type to CENP-A^CaCse4^ repressed conditions. A decrease in the stall signal is observed in *rad51* and *rad52* mutants as compared to the wild-type. The values represent the mean of three independent 2D experiments ± SD.
(TIF)

Figure S3 Rad51 or Rad52 depletion affects CENP-A^CaCse4^ localization at the centromere. Wild-type, *rad51* or *rad52* mutant cells were fixed and stained with DAPI (DNA) and anti-Prot A antibodies to study the localization of CENP-A^CaCse4^-Prot A in these strains. Merged DAPI and CENP-A^CaCse4^-Prot A images indicate altered CENP-A^CaCse4^ localization at the G2/M stages in *rad51* or *rad52* mutant strains as compared to the wild-type. Bar (white line), 5 µm.
(TIF)

Figure S4 CENP-A^CaCse4^ levels are reduced at the G2/M stage under depletion of Rad51 or Rad52. (A) The table shows the representative GFP-CENP-A^CaCse4^ image at each cell cycle stage in wild-type and *rad51* and *rad52* mutants along with the corresponding mean ± S.E.M (standard error of mean) values of GFP-CENP-A^CaCse4^ intensity. N = 10 for each cell cycle stage under different backgrounds. It is to be noted that the extended large bud phenotypes were included under the G2/M category in the *rad51* or *rad52* mutants. Bar (white line), 5 µm. (B) Normalized mean GFP-CENP-A^CaCse4^ intensity values were calculated and plotted for unbudded cells in wild-type, *rad51* and *rad52* mutants. (C) Normalized mean GFP-CENP-A^CaCse4^ intensity values were calculated and plotted for small budded cells in wild-type, *rad51* and *rad52* mutants. (D) Combined histogram of the GFP-CENP-A^CaCse4^intensity values for different cell cycle stages in wild-type, *rad51* and *rad52* mutants is shown.
(TIF)

Figure S5 Rad51 or Rad52 depletion affects Mis12^CaMtw1^ localization at the centromere. Wild-type, *rad51* or *rad52* mutant cells were fixed and stained with DAPI (DNA) and anti-Prot A antibodies to study the localization of Mis12^CaMtw1^-Prot A in these strains. Merged DAPI and Mis12^CaMtw1^-Prot A images indicate altered localization of the middle kinetochore protein Mis12^CaMtw1^ at the G2/M stages in *rad51* or *rad52* mutant strains as compared to the wild-type. Bar (white line), 5 µm.
(TIF)

Figure S6 Effect of Rad51 or Rad52 depletion on kinetochore protein recruitment and stability. (A) Standard ChIP assays were performed in wild-type, *rad51* and *rad52* (*MTW1/MTW1-TAP*) using anti-Prot A antibodies followed by PCR with primers that amplify *CEN5* or *CEN7*. Quantitative real time PCR (qPCR) of total DNA and with (+) antibody ChIP DNA fractions was performed. qPCR amplification from a non-centromeric control (non-*CEN*) was also performed to detect the background DNA elution in the ChIP assays. Enrichment of Mis12^CaMtw1^ at the centromere was calculated as a percentage of the total chromatin input. Values were plotted as mean of three biological replicates ± SD. (B) Western blot analysis was performed with whole cell lysates from wild-type, *rad51* or *rad52* mutants using anti-Prot A antibodies in order to detect the total protein levels of Mis12^CaMtw1^-Prot A. Actin and PSTAIRE are used as loading controls.(C) Total RNA was isolated from wild-type, *rad51* and *rad52* mutants. cDNA was prepared and RT (reverse transcriptase-PCR) was performed with primers specific to *CSE4* ORF. Left panel shows the RT-PCR levels of *CSE4* RNA in wild-type, *rad51* and *rad52* strains. *CDC28* was used as control. Right panel shows the RT-qPCR results of *CSE4* RNA in wild-type, *rad51* and *rad52* strains. The relative levels of *CSE4* RNA (*CSE4/CDC28*) were calculated and plotted as a mean of 2 biological replicates with samples by triplicates ± S.D. (D) Western blot analysis was performed with whole cell lysates from wild-type, *rad51* or *rad52* mutants using anti-Prot A antibody in order to detect the total protein levels of CENP-A^CaCse4^-Prot A or the non-degradable CENP-A^CaCse47R^-Prot A. PSTAIRE is used as the loading control. The relative levels of CENP-A^CaCse4^-Prot A or CENP-A^CaCse47R^-Prot A (CENP-A^CaCse4^/PSTAIRE) was computed for each mutant and plotted in a bar graph. (E) Standard ChIP assays followed by quantitative real time PCR (qPCR) were performed with anti-V5 antibody in Rad51-V5 and Rad52-V5 tagged strains for the central regions of *CEN7* and a non-*CEN* control. Enrichment of Rad51-V5/Rad52-V5 at the centromere was calculated as a percentage of the total chromatin input and values were plotted as mean of three biological replicates ± SD.
(TIF)

Table S1 List of strains and plasmids used in the study.
(DOC)

Table S2 List of oligonucleotide primers used in the study.
(DOC)
