# Supplementary figures and images for: Rad51–Rad52 Mediated Maintenance of Centromeric Chromatin in Candida albicans
Source: PLoS Genet. 2014 Apr 24;10(4):e1004344. doi: 10.1371/journal.pgen.1004344 (PMC3998917; doi:10.1371/journal.pgen.1004344)

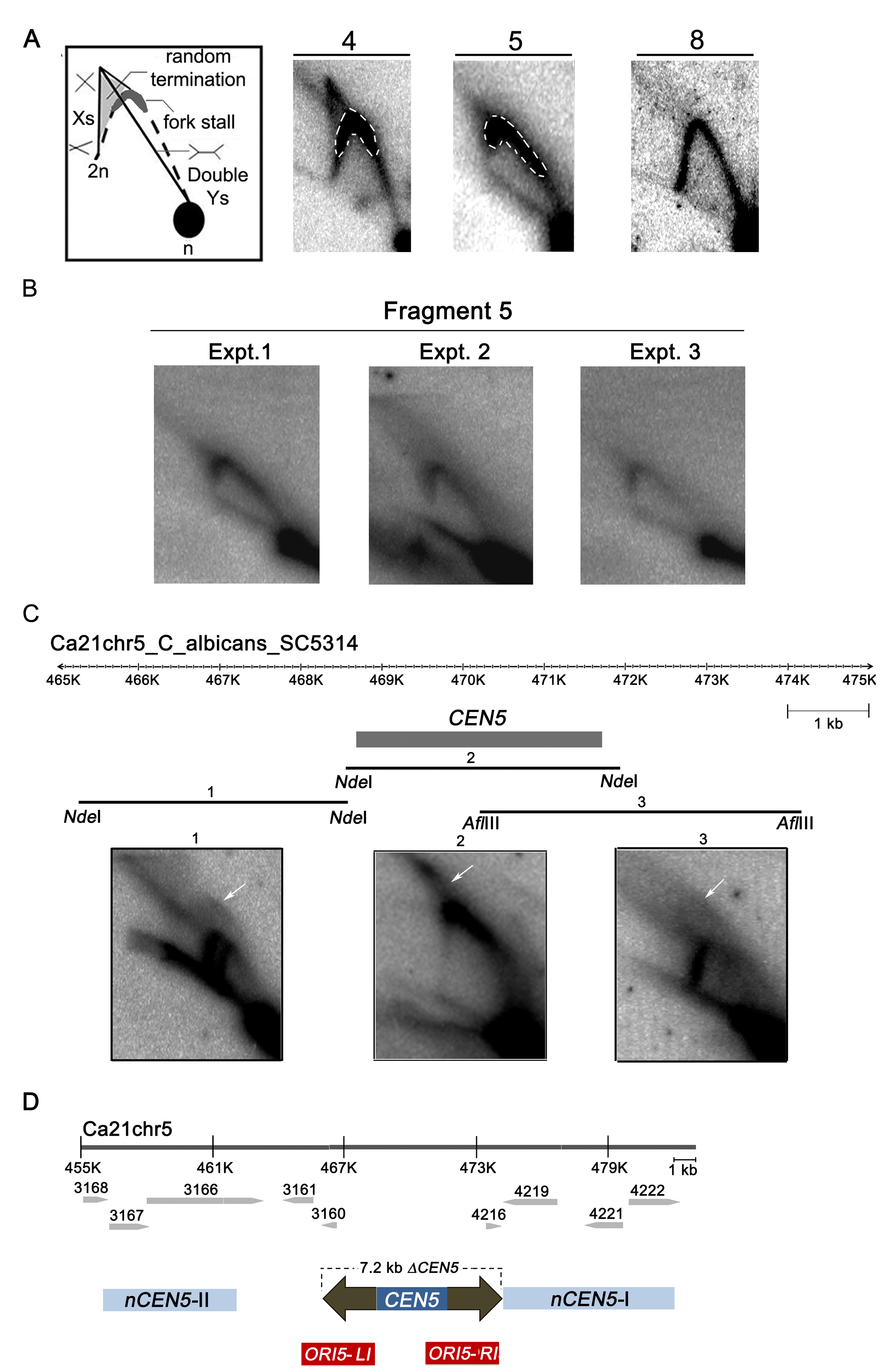

Supplement: Figure S1 — Analysis of fork stalling at C. albicans centromeres. (A) Schematics of replication intermediates as described in Figure 1. High contrast images of fragments 4, 5 and 8 of Figure 1 are reproduced in order to visualize the enhanced CEN7 stall signals. The stall signal is marginally expanded towards the ascending portion of the Y arc in fragment 5 as compared to fragment 4 (area bordered by white dotted lines). The blot containing fragment 4 when reprobed for fragment 8 does not show the intense signal at the inflection of the Y arc. (B) Three independent 2-D blots of the fragment 5 are shown in order to demonstrate the shift in the stall signal. (C) A line diagram of ∼10 kb region of chromosome 5 centered on the centromere (CEN5) is shown. The grey rectangle indicates CEN5. Restriction fragments (black lines 1–3) covering the upstream, core CEN5 and downstream regions are shown. On probing wild-type C. albicans genomic DNA (digested with the specified enzyme and separated by 2-D gel electrophoresis) with a unique region within CEN5, the 2-D blot shows the presence of both Y arcs as well as termination signals (white arrow in 2). Upstream and downstream fragments 1 and 3 show the presence of bubble arcs (white arrow in 1 and 3), indicating origins. (D) A line diagram of a ∼28 kb region around CEN5 along with the position of the ORFs (grey arrowhead). The corresponding positions of CEN5 and the nearest neocentromeres, nCEN5-I and nCEN5-II, are shown. Similarly, the positions of the origins ORI5-LI and ORI5-RI are indicated. The arrowheads (dark brown) represent the inverted repeats surrounding CEN5. (TIF) [file pgen.1004344.s001.tif]

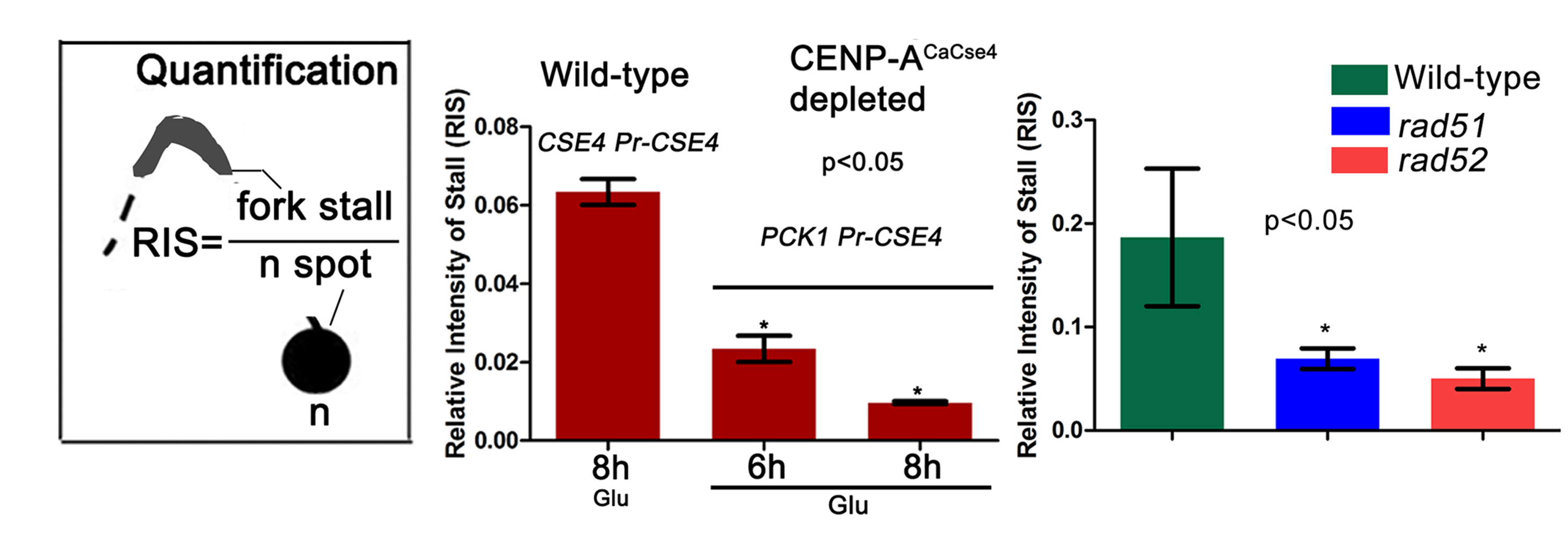

Supplement: Figure S2 — Fork stalling at C. albicans centromeres involves CENP-ACaCse4 and Rad51/Rad52. Quantification of the stall signal was performed as following: Relative intensity of stall (RIS) = fork stalling signal/1n spot. The 1n spot (schematic) and stall signals (intensity at the inflection of Y arc) were quantified by Image Gauge software (Fujifilm) and RIS values were calculated as described previously for wild-type vs CENP-ACaCse4 and wild-type vs Rad51/Rad52 depleted condition. The RIS values, plotted on a bar graph, indicate a gradual decrease in the stall signal from wild-type to CENP-ACaCse4 repressed conditions. A decrease in the stall signal is observed in rad51 and rad52 mutants as compared to the wild-type. The values represent the mean of three independent 2D experiments ± SD. (TIF) [file pgen.1004344.s002.tif]

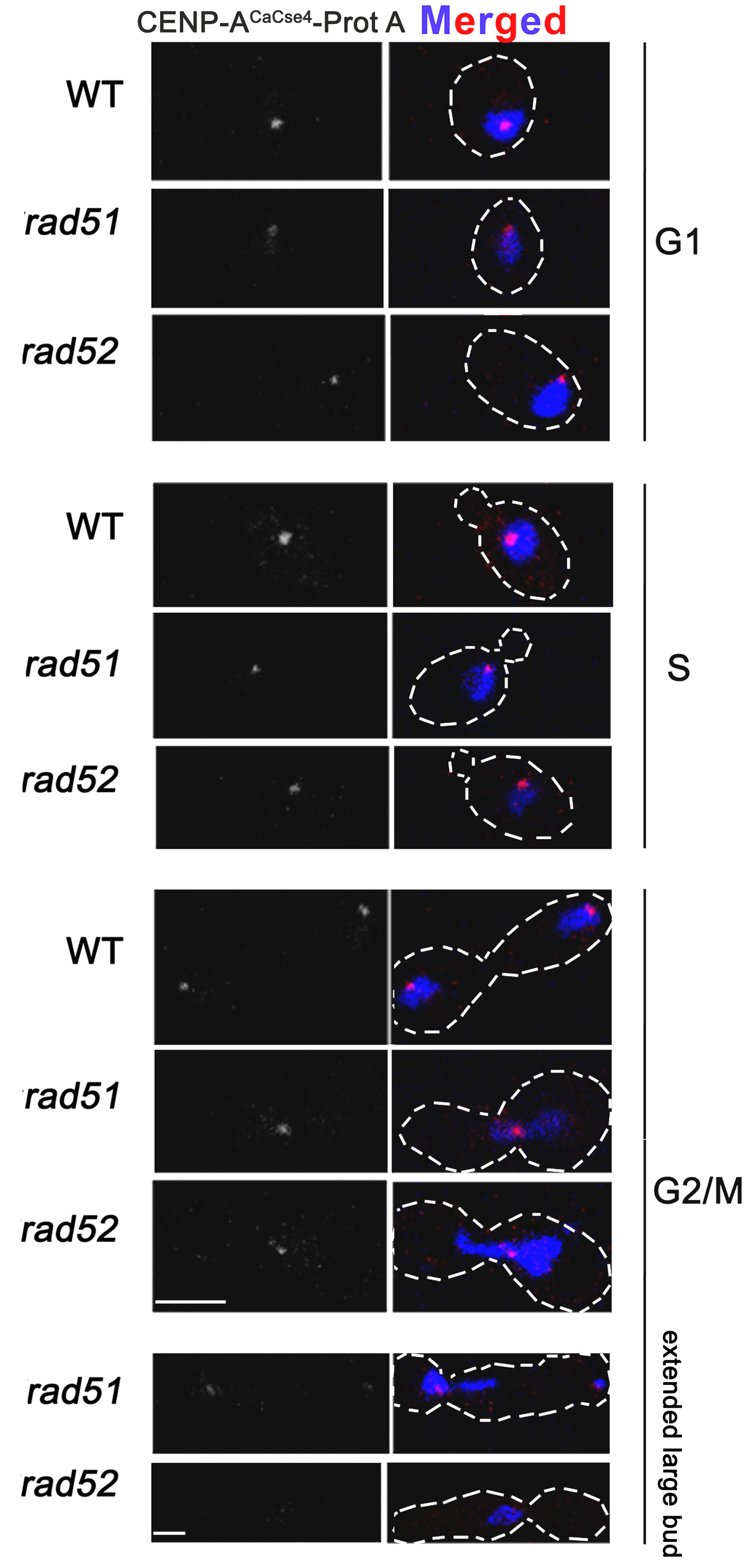

Supplement: Figure S3 — Rad51 or Rad52 depletion affects CENP-ACaCse4 localization at the centromere. Wild-type, rad51 or rad52 mutant cells were fixed and stained with DAPI (DNA) and anti-Prot A antibodies to study the localization of CENP-ACaCse4-Prot A in these strains. Merged DAPI and CENP-ACaCse4-Prot A images indicate altered CENP-ACaCse4 localization at the G2/M stages in rad51 or rad52 mutant strains as compared to the wild-type. Bar (white line), 5 µm. (TIF) [file pgen.1004344.s003.tif]

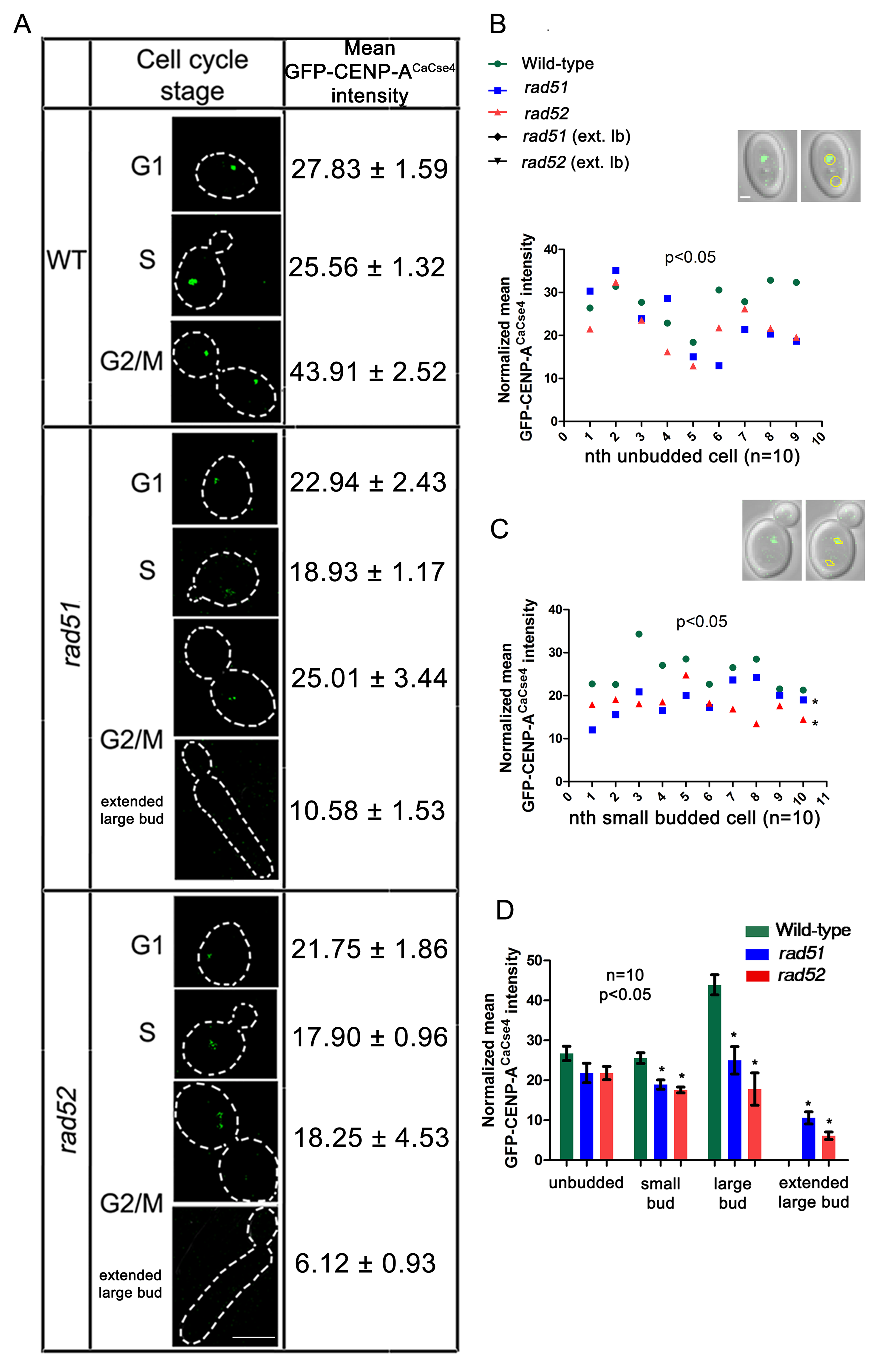

Supplement: Figure S4 — CENP-ACaCse4 levels are reduced at the G2/M stage under depletion of Rad51 or Rad52. (A) The table shows the representative GFP-CENP-ACaCse4 image at each cell cycle stage in wild-type and rad51 and rad52 mutants along with the corresponding mean ± S.E.M (standard error of mean) values of GFP-CENP-ACaCse4 intensity. N = 10 for each cell cycle stage under different backgrounds. It is to be noted that the extended large bud phenotypes were included under the G2/M category in the rad51 or rad52 mutants. Bar (white line), 5 µm. (B) Normalized mean GFP-CENP-ACaCse4 intensity values were calculated and plotted for unbudded cells in wild-type, rad51 and rad52 mutants. (C) Normalized mean GFP-CENP-ACaCse4 intensity values were calculated and plotted for small budded cells in wild-type, rad51 and rad52 mutants. (D) Combined histogram of the GFP-CENP-ACaCse4intensity values for different cell cycle stages in wild-type, rad51 and rad52 mutants is shown. (TIF) [file pgen.1004344.s004.tif]

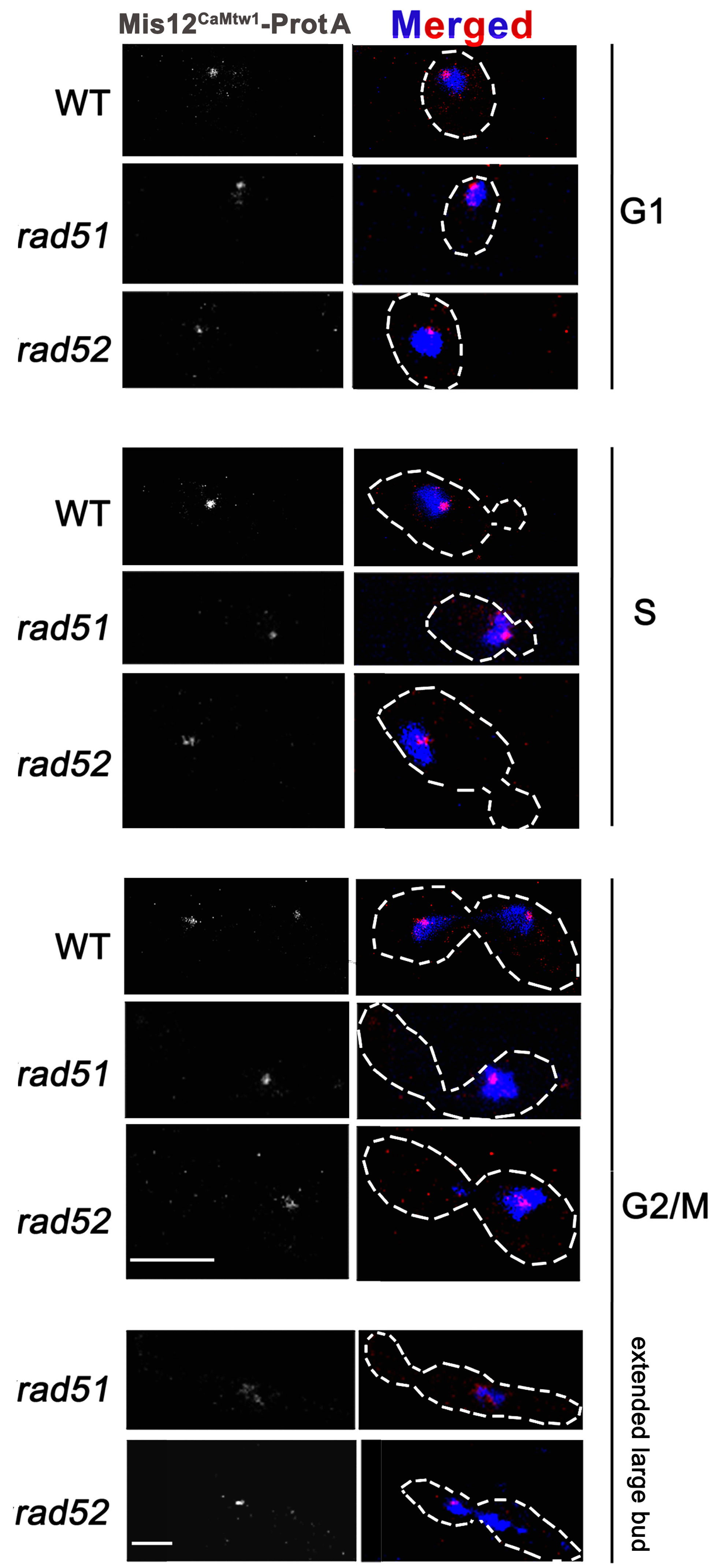

Supplement: Figure S5 — Rad51 or Rad52 depletion affects Mis12CaMtw1 localization at the centromere. Wild-type, rad51 or rad52 mutant cells were fixed and stained with DAPI (DNA) and anti-Prot A antibodies to study the localization of Mis12CaMtw1-Prot A in these strains. Merged DAPI and Mis12CaMtw1-Prot A images indicate altered localization of the middle kinetochore protein Mis12CaMtw1 at the G2/M stages in rad51 or rad52 mutant strains as compared to the wild-type. Bar (white line), 5 µm. (TIF) [file pgen.1004344.s005.tif]

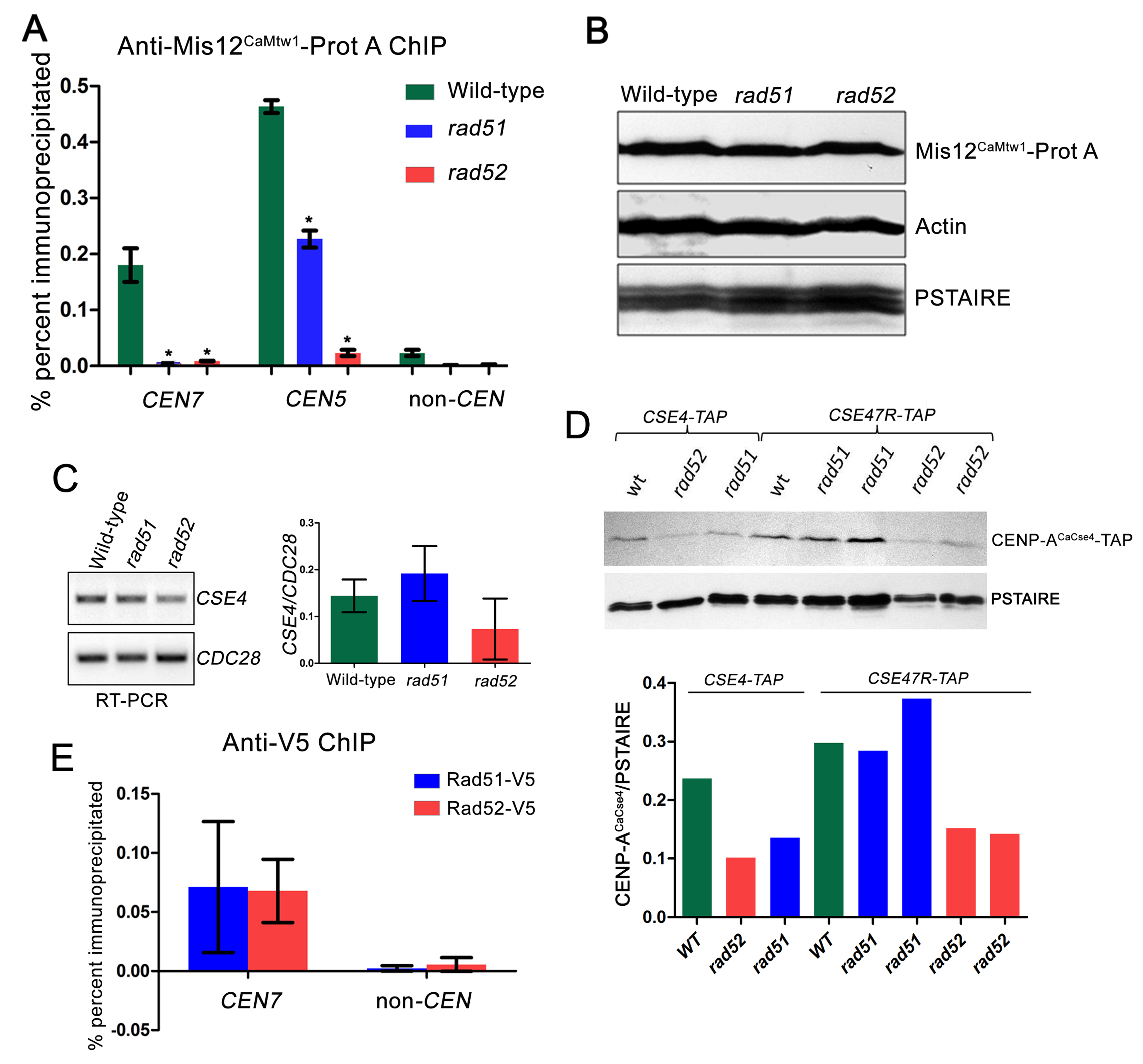

Supplement: Figure S6 — Effect of Rad51 or Rad52 depletion on kinetochore protein recruitment and stability. (A) Standard ChIP assays were performed in wild-type, rad51 and rad52 (MTW1/MTW1-TAP) using anti-Prot A antibodies followed by PCR with primers that amplify CEN5 or CEN7. Quantitative real time PCR (qPCR) of total DNA and with (+) antibody ChIP DNA fractions was performed. qPCR amplification from a non-centromeric control (non-CEN) was also performed to detect the background DNA elution in the ChIP assays. Enrichment of Mis12CaMtw1 at the centromere was calculated as a percentage of the total chromatin input. Values were plotted as mean of three biological replicates ± SD. (B) Western blot analysis was performed with whole cell lysates from wild-type, rad51 or rad52 mutants using anti-Prot A antibodies in order to detect the total protein levels of Mis12CaMtw1-Prot A. Actin and PSTAIRE are used as loading controls.(C) Total RNA was isolated from wild-type, rad51 and rad52 mutants. cDNA was prepared and RT (reverse transcriptase-PCR) was performed with primers specific to CSE4 ORF. Left panel shows the RT-PCR levels of CSE4 RNA in wild-type, rad51 and rad52 strains. CDC28 was used as control. Right panel shows the RT-qPCR results of CSE4 RNA in wild-type, rad51 and rad52 strains. The relative levels of CSE4 RNA (CSE4/CDC28) were calculated and plotted as a mean of 2 biological replicates with samples by triplicates ± S.D. (D) Western blot analysis was performed with whole cell lysates from wild-type, rad51 or rad52 mutants using anti-Prot A antibody in order to detect the total protein levels of CENP-ACaCse4-Prot A or the non-degradable CENP-ACaCse47R-Prot A. PSTAIRE is used as the loading control. The relative levels of CENP-ACaCse4-Prot A or CENP-ACaCse47R-Prot A (CENP-ACaCse4/PSTAIRE) was computed for each mutant and plotted in a bar graph. (E) Standard ChIP assays followed by quantitative real time PCR (qPCR) were performed with anti-V5 antibody in Rad51-V5 and Rad52-V5 tagged st [file pgen.1004344.s006.tif]
