# Supplementary material for: Rad51–Rad52 Mediated Maintenance of Centromeric Chromatin in Candida albicans
Source: PLoS Genet. 2014 Apr 24;10(4):e1004344. doi: 10.1371/journal.pgen.1004344 (PMC3998917; doi:10.1371/journal.pgen.1004344)
Supplement: Table S1 — List of strains and plasmids used in the study. (DOC) [file pgen.1004344.s007.doc]

Table S1. List of strains and plasmids used in the study.

| **Strain** | **Genotype** | **Reference** |
| --- | --- | --- |
| SC5314 | Wild-type | [74] |
| BWP17 | *Δura3::imm434/Δura3::imm434, Δhis1::hisG/Δhis1::hisG, Δarg4::hisG/Δarg4::hisG* | [65] |
| SN148 | *Δura3*::*imm434/Δura3*::*imm434 Δhis1::hisG/Δhis1::hisG, Δarg4::hisG/Δarg4::hisG,*  *Δleu2::hisG/Δleu2::hisG* | [75] |
| RM1000AH | *Δura3::imm434/Δura3::imm434Δhis1::hisG/ Δhis1::hisG arg4::HIS1/ARG4* | [33] |
| USN148 | *Δura3*::*imm434/Δura3*::*imm434/*CIp_10_  *Δhis1::hisG/Δhis1::hisG, Δarg4::hisG/Δarg4::hisG,*  *Δleu2::hisG/Δleu2::hisG* | This study |
| CAKS3b | *Δura3*::*imm434/Δura3*::*imm434,*  *cse4*::*PCK1*pr*-CSE4(URA3)/cse4*::*hisG* | [49] |
| YJB8675 | *Δura3*::*imm434/Δura3*::*imm434,*  *Δhis1*::*hisG/Δhis1*::*hisG Δarg4*::*hisG/Δarg4*::*hisG,*  *CSE4/CSE4-*GFP*-CSE4* | [48] |
| CAKS101 | *Δura3*::*imm434/Δura3*::*imm434,*  *Δhis1*::*hisG/Δhis1*::*hisG, Δarg4*::*hisG/Δarg4*::*hisG,*  *CSE4/CSE4-*GFP*-CSE4 Δrad52::hisG/rad52::hisG:URA3:hisG* | This study |
| GRC277 | *Δura3*::*imm434/Δura3*::*imm434,*  *Δhis1*::*hisG/Δhis1*::*hisG, Δarg4*::*hisG/Δarg4*::*hisG,*  *CSE4/CSE4-*GFP*-CSE4, rad51::HIS1/rad51::ARG4* | This study |
| CAKS102 | *Δura3*::*imm434/Δura3*::*imm434,*  *Δhis1::hisG/Δhis1::hisG, Δarg4::hisG/Δarg4::hisG,*  *Δleu2::hisG/Δleu2::hisG CSE4/CSE4-TAP(URA3)* | This study |
| GRC144 | *Δura3*::*imm434/Δura3*::*imm434*  *Δhis1::hisG/Δhis1::hisG Δarg4::hisG/Δarg4::hisG*  *Δleu2::hisG/Δleu2::hisG CSE4/CSE4-TAP(URA3)*  *rad52::HIS1/rad52::ARG4* | This study |
| GRC122 | *Δura3*::*imm434/Δura3*::*imm434,*  *Δhis1::hisG/Δhis1::hisG, Δarg4::hisG/Δarg4::hisG,*  *Δleu2::hisG/Δleu2::hisG, CSE4/CSE4-TAP(URA3)*  *rad51::HIS1/rad51::ARG4* | This study |
| CAKS106 | *Δura3*::*imm434/Δura3*::*imm434,*  *Δhis1::hisG/Δhis1::hisG, Δarg4::hisG/Δarg4::hisG,*  *Δleu2::hisG/Δleu2::hisG ,MTW1/MTW1-TAP(URA3)* | This study |
| GRC164 | *Δura3*::*imm434/Δura3*::*imm434,*  *Δhis1::hisG/Δhis1::hisG ,Δarg4::hisG/Δarg4::hisG,*  *Δleu2::hisG/Δleu2::hisG, MTW1/MTW1-TAP(URA3) rad52::HIS1/rad52::ARG4* | This study |
| GRC162 | *Δura3*::*imm434/Δura3*::*imm434,*  *Δhis1::hisG/Δhis1::hisG ,Δarg4::hisG/Δarg4::hisG,*  *Δleu2::hisG/Δleu2::hisG MTW1/MTW1-TAP(URA3), rad51::HIS1/rad51::ARG4* | This study |
| GRC68 | *Δura3*::*imm434/Δura3*::*imm434,RAD51-V5-URA3/rad51::hisG* | This study |
| GRC83 | *Δura3*::*imm434/Δura3*::*imm434,*  *Δhis1::hisG/Δhis1::hisG, Δarg4::hisG/Δarg4::hisG,*  *RAD52-V5-URA3/rad52::HIS1* | This study |
| JGR1.1 | *Δura3*::*imm434/Δura3*::*imm434, RAD51/rad51::hisG* | [76] |
| GRC55 | *Δura3*::*imm434/Δura3*::*imm434, Δhis1::hisG/Δhis1::hisG, Δarg4::hisG/Δarg4::hisG,RAD52/rad52::ARG4* | This study |
| CAKS105 | *Δura3*::*imm434/Δura3*::*imm434,*  *Δhis1::hisG/Δhis1::hisG ,Δarg4::hisG/Δarg4::hisG,*  *Δleu2::hisG/Δleu2::hisG, CSE4/CSE4-TAP (HIS1), ∆ORI7-RI::URA3/∆ORI7-R1::NAT* | This study |
| CAKS104 | *Δura3::imm434/ Δura3::imm434 Δhis1::hisG/ Δhis1::hisG arg4::HIS1/ARG4,ORI7-RI/∆ORI7-RI::ura3* | This study |
| CAKS103 | *Δura3*::*imm434/Δura3*::*imm434, Δhis1::hisG/Δhis1::hisG, Δarg4::hisG/Δarg4::hisG, Δleu2::hisG/Δleu2::hisG CSE4/CSE4-TAP(HIS1)* | This study |
| J129 | *∆ura3::imm434/∆ura3::imm434∆his1::hisG/∆his1::his*  *G∆arg4::hisG/∆arg4::hisG CSE47R-TAP(URA3)/cse4::hisG* | [44] |
| GRC409 | *∆ura3::imm434/∆ura3::imm434∆his1::hisG/∆his1::his*  *G∆arg4::hisG/∆arg4::hisG CSE47R-TAP(URA3)/cse4::hisG;rad51::ARG4/rad51::HIS1* | This study |
| GRC425 | *∆ura3::imm434/∆ura3::imm434∆his1::hisG/∆his1::his*  *G∆arg4::hisG/∆arg4::hisG CSE47R-TAP(URA3)/cse4::hisG;rad52::ARG4/rad52::HIS1* | This study |
| CAI-4 | *ura3::imm434/ura3::imm434* | [77] |
| JGR5A | *ura3::imm434/ura3::imm434, rad51::hisG/rad51::hisG* | [76] |
| TCR2.1.1 | *ura3::imm434/ura3::imm434, rad52::hisG/rad52::hisG* | [78] |
| BNC23.1 | *ura3::imm434/ura3::imm434, rad59::hisG/rad59::hisG* | [76] |
| LCD2A1 | *ura3::imm434/ura3::imm434, ku70::hisG/ku70::hisG* | [79] |
| DKCa58 | *arg4/arg4 his1/his1 ura3::imm434/ura3::imm434, GAL1/gal1:URA, mre11 ::HIS1/mre11 ::ARG4* | [80] |
| DKCa67 | *arg4/arg4 his1/his1 ura3::imm434/ura3::imm434, GAL1/gal1:URA, rad50 ::HIS1/rad50 ::ARG4* | [80] |
| DKCa78 | *arg4/arg4 his1/his1 ura3::imm434/ura3::imm434, GAL1/gal1:URA, yku80 ::HIS1/yku80 ::ARG4* | [80] |
| **Plasmid** | **Description** | **Reference** |
| pKS101 | pUC19 + CaURA3 | This study |
| p*ORI7-RI* | pKS101 + 1.4 kb intergenic region from *ORI7-RI* | This study |
| p*ORI7-LI* | pKS101 + 2.4 kb intergenic region from *ORI7-LI* | This study |
| pMTU2 | pBluescript-URA3 + C term. MTW1 + TAP + 3'MTW1 UTR | [50] |
| pMG2090 | pBluescript +V5 + TADH1 + CaURA3 | [81] |
| pCIp10 | pBluescript + RP10 + CaURA3 | [66] |

**References**

74. Gillum AM, Tsay EY, Kirsch DR (1984) Isolation of the Candida albicans gene for orotidine-5'-phosphate decarboxylase by complementation of S. cerevisiae ura3 and E. coli pyrF mutations. Mol Gen Genet 198: 179-182.

75. Noble SM, Johnson AD (2005) Strains and strategies for large-scale gene deletion studies of the diploid human fungal pathogen Candida albicans. Eukaryot Cell 4: 298-309.

76. Garcia-Prieto F, Gomez-Raja J, Andaluz E, Calderone R, Larriba G (2010) Role of the homologous recombination genes RAD51 and RAD59 in the resistance of Candida albicans to UV light, radiomimetic and anti-tumor compounds and oxidizing agents. Fungal Genet Biol 47: 433-445.

77. Fonzi WA, Irwin MY (1993) Isogenic strain construction and gene mapping in Candida albicans. Genetics 134: 717-728.

78. Ciudad T, Andaluz E, Steinberg-Neifach O, Lue NF, Gow NA, et al. (2004) Homologous recombination in Candida albicans: role of CaRad52p in DNA repair, integration of linear DNA fragments and telomere length. Mol Microbiol 53: 1177-1194.

79. Chico L, Ciudad T, Hsu M, Lue NF, Larriba G (2011) The Candida albicans Ku70 Modulates Telomere Length and Structure by Regulating Both Telomerase and Recombination. PLoS One 6: e23732.

80. Legrand M, Chan CL, Jauert PA, Kirkpatrick DT (2007) Role of DNA mismatch repair and double-strand break repair in genome stability and antifungal drug resistance in Candida albicans. Eukaryot Cell 6: 2194-2205.

81. Gerami-Nejad M, Dulmage K, Berman J (2009) Additional cassettes for epitope and fluorescent fusion proteins in Candida albicans. Yeast 26: 399-406.
