# Supplementary material for: Rad51–Rad52 Mediated Maintenance of Centromeric Chromatin in Candida albicans
Source: PLoS Genet. 2014 Apr 24;10(4):e1004344. doi: 10.1371/journal.pgen.1004344 (PMC3998917; doi:10.1371/journal.pgen.1004344)
Supplement: Table S2 — List of oligonucleotide primers used in the study. (DOC) [file pgen.1004344.s008.doc]

Table S2. List of oligonucleotide primers used for the study.

| RAD51D-F | ACTTCAGCATCAGCGTTGATATTTTGAGGATGTGATCCTTCTTCATGAAGATCAACTTGTTCAATTTCTGTTTGAGTCATTTTCCCAGTCACGACGTT |
| --- | --- |
| RAD51D-R | CTACTCATTATCTTCAACTTTTGGATCACCAATACCGTCTTCGTATATGGCAAAGATACATTCACTTTCAGGCAAACAGGGTGGAATTGTGAGCGGATA |
| RAD52D-F | ATGAACTCTAGACCTGCACCTCCGCAACCACGACCACCACAACAACAACCTCAGCAACCTCAGCAACCTCAACCCAACCATTTCCCAGTCACGACGTT |
| RAD52D-R | TTATTGGTTAACAGTCGTATTAGCTATTGTAGAATTATTTTCAACATTCTCATTACCACTTGCACCATTAACAGAAGTAGGTGGAATTGTGAGCGGATA |
| RAD51det-F | AAAGGCAAGAATGAGATAAGAATA |
| RAD51det-R1 | GTGAAACCCAAGGGAACTAAT |
| RAD52det-F | ACAAACCCTTCCACCCACACT |
| RAD52det-R1 | CCCTACTGAAAATTTACCGCTACC |
| HIS1det-R | CTGGGATATCAGCTGCAGGC |
| ARG4det-R | GGAATTGATCAATTATCTTTTGAAC |
| RAD51F2 | GCCTGAAAGTGAATGTATCTTTGCCATATACGAAGACGGTATTGGTGATCCAAAAGTTGAAGATAATGAGGGTGGTGGTGGTAAGCCTATCCCTAACCCTCTC |
| RAD52F2 | TGTTAATGGTGCAAGTGGTAATGAGAATGTTGAAAATAATTCTACAATAGCTAATACGACTGTTAACCAAGGTGGTGGTGGTAAGCCTATCCCTAACCCTCTC |
| RAD51R1 | TGAACAACGATAATTTTTCAAAGTTTTCAACTTTGGGATAGTACAAACAAACATTTAAAACAATAATAATTCTAGAAGGACCACCTTTGATTG |
| RAD52R1 | ATAGATTAATCTACAACTAATCAAAATCTAATTGTAATATTAGCTTATTCCACAATTCTAGATACCTATATCTAGAAGGACCACCTTTGATTG |
| V5det-R | AGGAGAGGGTTAGGGATAGG |
| RAD51det-F1 | GCGTGCTTACAATGCTGAACA |
| RAD51det-R3 | ACCACGGGCTACTGAAAATCTC |
| RAD52det-F1 | ATGGGGAATCGCAGAGTA |
| RAD52det-R3 | AATGGTTTGGGTTATAGGATGAAT |
| SM-1 | GCCTCTTCTTCAACCCATT |
| SM-2 | CAT CAA TAA CCC TCT TGG CTC |
| SM-3 | ACTCGCCTTCCCCTCCTTTAAATAG |
| SM-4 | CCACTACTACGACTGTGGATTCACT |
| SM-5 | CGAGCTCGGCTAATCTCCTGTACAGTGG |
| SM-6 | GGGGTACCCCTGTGTTAGAGTACTTCACCC |
| SM-7 | CGAGCTCGCTTCTAACTGAAGCCCAAAA |
| SM-8 | GGGGTACCCCTTGATGGTAAGGTAAGGTATCGATC |
| SM-9 | TCCCCCGGGCGTCTGTTTCAGAGTCTGG |
| SM-10 | TCCCCCGGGGTCTCGATCTCACCAATAG |
| SM-11 | AGACAGCGAGCACATCATTAACCG |
| 2498-5 | GCGTAACGGGCCTAGTTTCGATAAGAG |
| 2498-9 | GAAACGATCCTTCCTGTACACCAC |
| 2498-22 | CCTGACACTGTCGTTTCCCATAGC |
| 2498-7 | GCCTGTAGCGATGTAAGTATATGGAG |
| 2498-8 | CCACCTCTGCACTAATCTACAATGC |
| 2498-17 | GCTTGGCCCTCAGTATAACTGGAT |
| 2498-18 | CTTCAGGACAAGCTCCATATCTCTTC |
| 2498-19 | GCCATACGGTAGTCAAACTCCTGG |
| 2498-20 | CCTGAACCACTACTGCAGAAACGT |
| 2498-21 | CTAGTGCAAGACCCTCATAGAAGC |
| 2498-15 | CAAGCTGCCTTGTCAGGCAAAGCATC |
| 2498-16 | CCATCTCCAACCCGCCATGCCAGC |
| 2498-24 | CAG AGC AAT GGC CCT TGT GAT TGT |
| CaChr7S13 | TCCACCTCCACCTCCACCT |
| CaChr7AS14 | ACCTCGACATCGACTTGATTGA |
| CaChr7S10 | TTGTTAGCATTGTTGTTGTTGTTGT |
| CaChr7AS11 | CGGTATTTCTGAGTGAATTAAAAAG |
| CaChr7S5 | GGACGGTAAATAGGTTAATGGAG |
| CaChr7AS6 | CGAGTTGTTATAGTTTGAATAAGCG |
| SM-12 | CAATGGAACGGTTATCACTT |
| SM-13 | AGCTGGTTTGTGAGTTAGGA |
| SM-14 | CAACTTCAAGGGTAATTTGG |
| SM-15 | TTCGGTGCCTACTTTACATT |
| SM-16 | AAAGAGCAGTTTCAGATCCA |
| SM-17 | TCAACCGGATATTGTCTACC |
| SM-18 | TGGTACCGATTAGCCTTCTA |
| SM-19 | GACCAATTATGTTCCTTCCA |
| SM-20 | GGTCCCTACGTTCGTCAAAA |
| SM-21 | TTTACTAGAGACCCGCACCAA |
| SM-22 | TTCTGCACTTGCTCAGATGG |
| SM-23 | TCCTTCGAATGCTTGTTTGA |
| nCEN7-1 | CACCTCTGCACTAATCTACAATG |
| nCEN7-2 | TGTTGAGATGTCTTATTGATTAGGT |
| nCEN7-5 | TCAATTATCGCTTGATAGCG |
| nCEN7-6 | CTATCATCATGCCAGCCTAG |
| SM-24 | CGGAAACGACCAGACTTGAT |
| SM-25 | GGTATCTCTGCCACCGATGA |
| nCEN7-3 | GCATACCTGACACTGTCGTT |
| nCEN7-4 | AACGGTGCTACGTTTTTTTA |
| CACH5F1 | CCCGCAAATAAGCAAACACT |
| CACH5R1 | TTCATGGAAGAGGGGTTTCA |
| nLeu2-1 | GTACCGAAATTGTCAATGAAG |
| nLeu2-2 | GTGGTGTTTGAAATCAAATTG |
| SM-26 | GGA ATT CCT GAT GCT TTG CCT GAC AAG |
| SM-27 | TTC CTA TTA TCC GGA AGC TTC CAA AGA AAA CAT CGA GAT |
| SM-28 | ATC AAA GGT GGT CCT TCT AAT TCT CTC AAT GTA AAC TGG A |
| SM-29 | GTC TAA TCC AAA TAC CAA TGC CGG GAT CCC G |
| SM-30 | ATC TCG ATG TTT TCT TTG GAA GCT TCC GGA TAA TAG GAA |
| SM-31 | CAC CTT TGA TTCC AGT TTA CAT TGA GAG AAT TAG AAG GAC |
| SM-32 | CGA TAC TAA CGC CGC CAT CCA GTT CCA AAG AAA ACA TCG AGA T |
| SM-33 | GGG GAC GAG GCA AGC TTG ATA TCT TCT CTC AAT GTA AAC TGG A |
| SM-34 | ATC TCG ATG TTT TCT TTG GAA CTG GAT GGC GGC GTT AGT ATC G |
| SM-35 | TCC AGT TTA CAT TGA GAG AAG ATA TCA AGC TTG CCT CGT CCC C |
| SM-36 | GAA AAG AGA AGA TGG AAA AAG AAT |
| SM-37 | TCC GTC TAC CGG GGT TTT CCT ATT TAC AAT CAA AGG TGG T |
| SM-38 | ACC ACC TTT GAT TGT AAA TAG GAA AAC CCC GGT AGA CGG A |
| SM-39 | ATA GTC TAT TCC AAA TCG TGG CCT CGT TTG TGA GAA ACC AA |
| SM-40 | TTG GTT TCT CAC AAA CGA GGC CAC GAT TTG GAA TAG ACT AT |
| SM-41 | TCT GTA TTT GCA AAC CAC TGA A |
| SM-42 | GAT TCA CGC CAA AAG AGT GAC TAT CAT GCA AAA GGA TAT ACA ATT AGC AAG AAG GAT CAG AGG ACA GTC TTG GAT ATT G GGA TCC ATG GAA AAG AGA AGA TGG AAA AAG |
| qCDC28-F | GTTATCTGATTATCAACGTCAAGAAAA |
| qCDC28-R | TCTAATGCTTTATAAACAACCCCATA |
| CDC28-F | GGCCTGATGTTAATTATTTACCAG |
| CDC28-R | GCATGTTTTGGTGTTTGTCAATC |
| CSE4-F | CTGGCCAAGGAACAAGTGC |
| CSE4-R | TGGCCCAACAAAATCAAGAC |
| qCse4-F | TGGCAAGACTTTCAGGACAA |
| qCse4-R | CCTCTGCACTTGTTCCTTGG |
